# Probiotic Development Strategy Centered on Stability and Regulatory Considerations

**DOI:** 10.1111/1541-4337.70320

**Published:** 2026-01-05

**Authors:** Hye Kim, Ariful Haque, Abdur Razzak, Min Ji Jang, Sehyeon Song, Seockmo Ku

**Affiliations:** ^1^ Department of Food Science and Technology Texas A&M University College Station Texas USA

**Keywords:** microencapsulation, probiotics, regulatory frameworks, stability, strain development

## Abstract

The development of probiotic strains has become a major focus in both academic and industrial research, driven by their health benefits and growing consumer demand. However, functional outcomes demonstrated under laboratory conditions often fail to align with the stability and large‐scale performance required for industrial applications, creating a major obstacle to commercialization. This gap underscores the need for standardized evaluation frameworks that integrate scientific validation with industrial performance metrics. This review critically examines the probiotic development pipeline, encompassing strain screening, resilience‐enhancing strategies, and global regulatory frameworks. Particular attention is given to the effects of production stressors such as heat, oxygen, and digestion on strain viability. Studies reporting up to a 31‐fold increase in survival after heat shock and up to a 100‐fold improvement through microencapsulation during drying are highlighted to illustrate both the potential and limitations of adaptive strategies. These findings reveal the strong strain specificity and inconsistent reproducibility of current approaches, offering important insights for strategic development. Furthermore, regulatory systems in the United States, European Union, Japan, Korea, and China are compared to emphasize how heterogeneity in classification, safety assessment, and functional substantiation complicates global market entry. This review delves into how harmonized evaluation frameworks and sustained collaboration between academia, industry, and regulatory authorities help to develop next‐generation probiotics by integrating functionality, safety, stability, industrial application, and regulation parameters to achieve balanced progress in efficacy, safety, scalability, and economic feasibility.

## 1 Introduction

Probiotic research has attracted growing academic attention due to its therapeutic potential in managing a broad spectrum of health conditions. In order to better understand the current research landscape and identify persisting gaps, we systematically analyzed recent review articles and categorized their contents into five major domains: functionality, safety, stability, industrial application, and regulation. Most studies extensively discussed the health‐promoting properties of probiotics, including gastrointestinal, metabolic, immunological, and neurological benefits, while also noting the limited standardization of strain‐specific functional indices and the shortage of long‐term and large‐scale clinical evidence (da Silva et al. 2024; Ge et al. 2024; Shah et al. 2024; Petrariu et al. 2024). Safety‐related discussions were largely framed within international regulatory guidelines such as Food and Agriculture Organization of the United Nations and the World Health Organization (FAO/WHO), Food and Drug Administration (FDA) generally recognized as safe (GRAS)/new dietary ingredient (NDI), and European Food Safety Authority (EFSA) QPS, with emphasis on genomic identification, toxin and antibiotic resistance gene screening, and particular caution in vulnerable populations. However, robust safety data for next‐generation probiotics (NGPs) remain scarce, and globally harmonized criteria are still lacking (Roe et al. 2022; Spacova et al. 2023; Liang et al. 2024). In addition, stability, defined as the maintenance of probiotic viability during processing, storage, and delivery, was primarily addressed in the context of encapsulation technologies, protective agents, and stress adaptation strategies, although practical improvement methods validated under real industrial settings are rare, and long‐term preservation data at industrial scale remain scarce (Lin, Si, et al. 2024; Ge et al. 2024; Wang and Zhong 2024). From an industrial perspective, advancements in omics‐guided strain optimization, bioreactor cultivation, incorporation into diverse food matrices, and drying technologies for improved viability have been reported, but critical analyses of successful commercialization cases and economic feasibility evaluations are still insufficient (Yang et al. 2024; Pereira et al. 2024; Liang et al. 2024). Finally, the regulatory landscape is marked by pronounced heterogeneity across regions, limited harmonization, and no clear roadmaps for next‐generation or genetically engineered strains. Comparative evaluations of regulatory commonalities and differences are rarely provided, concise summaries of mandatory evaluation requirements are lacking, and the economic and temporal burdens imposed by divergent regulatory frameworks remain underexplored (Roe et al. 2022; Spacova et al. 2023; Aziz and Zaidi 2025; da Silva et al. 2024). Collectively, this comparative synthesis highlights both the significant progress in probiotic science and the persistent gaps in scientific validation, consumer safety, and industrial scalability. These gaps underscore the necessity of a focused review that not only synthesizes prior findings but also addresses unresolved issues of particular importance. A comparative summary of how recent review articles have addressed five major domains is provided for reference (Table S1). Importantly, this article explicitly situates itself in relation to the most frequently cited earlier reviews, including those addressing probiotic health benefits (Sarita et al. 2025), safety perspectives (Roe et al. 2022), regulatory challenges (Aziz and Zaidi 2025), and evolutionary adaptation (Leeflang et al. 2025), as well as a broader set of recent publications.

**FIGURE 1:**
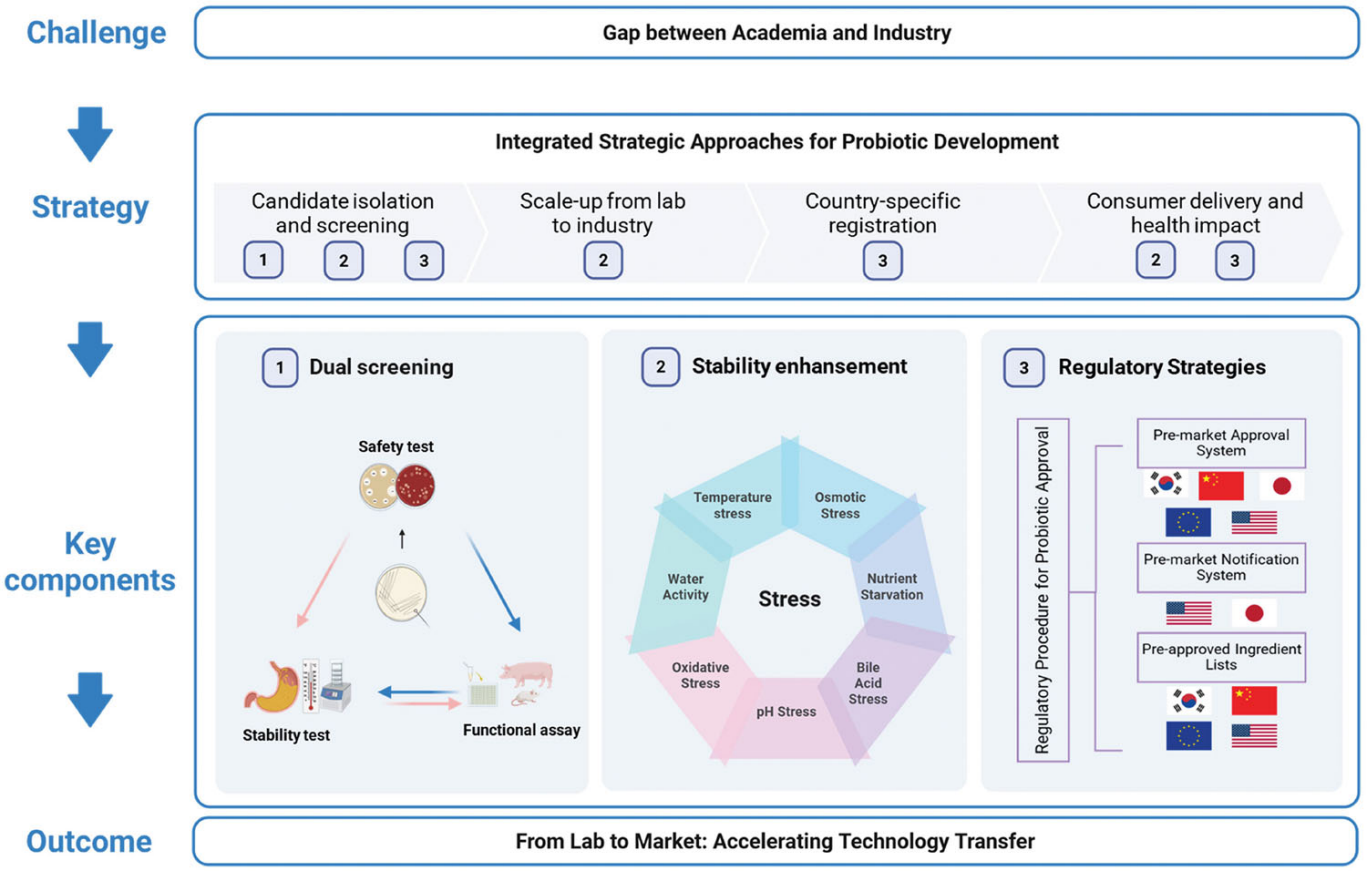
Integrated strategic framework for probiotic development and commercial translation.

Building on these identified gaps, the present review examines the entire continuum that connects academia and industry, from strain isolation to commercialization, and highlights the most critical stage of early strain selection by providing a practical guideline for efficient candidate screening. In addition, it discusses several key issues that have not been sufficiently covered in previous literature. These include the shortage of long‐term and large‐scale clinical evidence in the functionality domain, the lack of robust safety data for NGPs, the scarcity of stability improvement strategies validated under industrial conditions and the limited critical analyses of existing commercialization and preservation cases, insufficient critical analyses of successful commercialization cases, inadequate comparative evaluations of regulatory frameworks, the absence of concise summaries of mandatory evaluation requirements, and the heavy economic and temporal burdens caused by heterogeneous regulations. These topics represent both the research gaps left by prior reviews and the targeted focus of the present article (Figure 1).

Collectively, these basic research studies underscore the expanding application spectrum of probiotics and emphasize their mechanisms and clinical efficacy. In contrast, industrial probiotic development is primarily driven by consumer preferences and market feasibility. Unlike industry, which faces strong time‐to‐market pressures, academic research operates in a more flexible environment that allows sustained creativity and persistence without the immediate burden of commercialization. This unique context fosters the emergence of highly innovative and functionally promising strains, a perspective that prior reviews have seldom addressed (Masclans et al. 2025). However, to bring such academically discovered strains successfully to market, rigorous stability and safety considerations must be integrated early in development. This approach holds great promise for maximizing their eventual benefits to human health. Product innovation in the commercial sector focuses heavily on the diversification of delivery formats to include capsules, powders, gummies, fermented dairy products, and functional beverages, all aimed at consumer acceptability and convenience. Along with the establishment of formulation strategies, market entry relies on the pursuit of regulatory certifications, such as GRAS and NDI status. Marketing practices often center on broadly appealing health claims, including “immune support” and “digestive health,” which are readily recognized by consumers, despite varying levels of scientific substantiation. Furthermore, to accelerate commercialization, many industry efforts prioritize speed‐to‐market and cost efficiency over comprehensive scientific validation. Recent analyses indicate that companies tend to select research outputs that are directly linked to patents or that demonstrate immediate applicability, reflecting structural pressures that favor commercial utility over scientific rigor (Masclans et al. 2025). Consequently, industry‐driven studies tend to emphasize perceptible outcomes, such as improved bowel function or relief from constipation, and they often rely on pre‐approved strains and observational data.

This approach, while practical, can limit scientific rigor and reduce acceptance in peer‐reviewed academic settings. Moreover, functional strains identified through academic research frequently exhibit poor industrial stability, particularly during manufacturing processes, such as heating, drying, and storage. Maintaining their viability often requires specialized protective matrices, optimized formulations, and cold‐chain logistics, all of which increase production costs and reduce consumer accessibility. Compounding these challenges, insufficient viable cell counts in final commercial products can compromise functional efficacy and consumer trust, whereas the absence of an effective translational pipeline between academic discovery and industrial application contributes to delays in product development. Cooperative education programs and research internships have emerged to bridge this divide (Castelló et al. 2023; Martirosyan and Alvarado 2023), but systematic frameworks for technology transfer remain underdeveloped.

**FIGURE 2:**
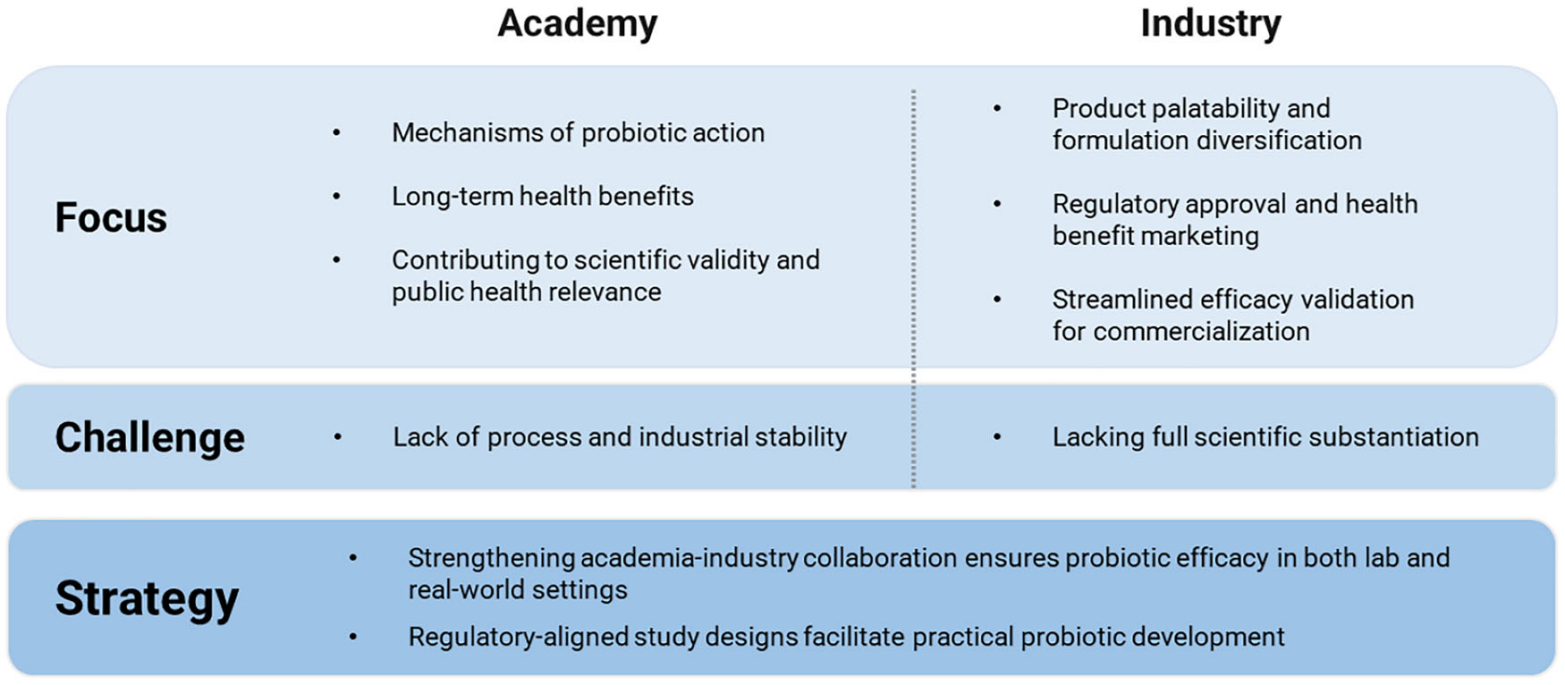
Bridging academic and industrial perspectives in probiotic development.

To address these issues, two strategic directions are essential to move probiotics research forward (Figure 2). First, stronger academia‐industry collaboration is crucial to ensure that probiotic functionality is verified both under laboratory conditions and within real‐world product environments. Key attributes, such as formulation design, gastrointestinal survivability, and storage stability, must be jointly evaluated to align scientific findings with consumer‐level outcomes. Specific strategies addressing these issues are presented in Sections 2 and 3 of this review. Second, given the absence of globally harmonized regulatory standards, aligning experimental designs with country‐specific requirements has become a practical and necessary strategy for the development of probiotic products destined for international markets. Although the FAO and WHO have established foundational safety and efficacy criteria in their “Guidelines for the Evaluation of Probiotics in Food” (Araya et al. 2002), current regulatory frameworks remain highly heterogeneous across countries and institutions. This variation does not necessarily imply that the same scientific data are interpreted differently, but rather that distinct threshold values and evaluation criteria are applied by different authorities. Consequently, identical experimental outcomes may yield divergent regulatory decisions, as a result complicating product development and delaying market entry. In summary, regulatory safety evaluation requirements profoundly shape experimental design and create substantial barriers to global commercialization, underscoring their critical impact on probiotic research and industry.

## 2 Selection of Lead Probiotics Strains

The development of lead probiotic strains, as a fundamental component of the probiotics industry, has as its aim the identification of microorganisms that demonstrate high functional efficacy while remaining compatible with industrial processing conditions. In this context, the initial stages of strain isolation and screening are critically important, as they often determine the ultimate success of the development pipeline. The development process typically begins with the isolation of potentially beneficial microorganisms from natural sources, such as the human gut microbiota, fermented foods, and breast milk. After isolation, candidate probiotic strains are subjected to a series of comprehensive assessments to establish essential factors, such as their safety, functional properties, and stability, using screening processes that generally involve the evaluation of several key characteristics, including acid and bile tolerance, antimicrobial activity, adhesion to intestinal epithelial cells, and metabolic capabilities.

### 2.1 Safety Evaluation

The most critical component in the safety screening of probiotic candidates is the assessment of antibiotic resistance, which is often considered one of the most stringent exclusion criteria in early stage strain development. These regulatory requirements have a direct impact on the probiotic pipeline, as a considerable number of candidate strains are eliminated at this stage, leading to reduced development efficiency but ensuring compliance with safety standards. In practice, a considerable number of candidate strains are eliminated at this stage due either to minimum inhibitory concentrations (MICs) that exceed regulatory thresholds or the presence of transferable resistance genes. Consequently, very early implementation of antibiotic resistance testing is strongly recommended to reduce resource waste and prioritize strains with a higher likelihood of meeting regulatory safety standards. A further commonly employed test in the initial safety evaluation is the hemolytic activity assay. Although other assessments, such as toxin production and detection of virulence‐associated genes, are also required at later stages, prioritizing antibiotic resistance and hemolysis testing in the early screening phase enhances development efficiency and regulatory preparedness.

Antibiotic resistance assessment is a mandatory safety criterion in probiotic development. Its purpose is to determine whether the candidate strain harbors antibiotic‐resistance genes or has the potential to transfer them to other microbes, raising the risk of horizontal gene transfer. Typically, the MIC values are compared against threshold levels provided by various authorities, such as the EFSA. The EFSA criteria are specifically tailored to probiotic microorganisms and are widely adopted in the probiotics field. If a strain exhibits MIC values above the established cutoff, molecular analyses must be conducted to confirm the presence of resistance genes. Any indication that these genes are plasmid‐borne necessitates further investigation due to the increased risk of horizontal transfer to pathogenic species. Accordingly, strains that surpass the MIC threshold should preferably be eliminated at the initial screening stage to ensure compliance with safety standards.

The probiotic strains themselves must also be free from pathogenic traits if they are intended for human use. Their hemolytic activity is a key indicator of their potential pathogenicity, and growth on blood agar plates is used to classify strains according to α‐, β‐, or γ‐hemolysis. The β‐hemolytic strains completely lyse red blood cells and are therefore considered potentially pathogenic and are typically disqualified. Conversely, γ‐hemolytic strains display no hemolytic activity and are regarded as relatively safe. The α‐hemolytic strains, which are characterized by partial hemolysis (indicated by a greenish discoloration around the colonies due to the oxidation of hemoglobin to methemoglobin), are not as hazardous as β‐hemolytic strains but still raise safety concerns and require careful evaluation. Thus, hemolysis assays serve as rapid and effective tools for identifying potential pathogenicity risks during the initial safety screening phase of a potential functional probiotic.

Beyond these conventional safety assessments, a persistent gap in the field is the scarcity of robust safety data for NGPs, including engineered strains and those originating from novel taxa (Vallianou et al. 2023). Unlike well‐characterized lactic acid bacteria (LAB) or bifidobacteria, NGPs often lack comprehensive long‐term safety evaluations, particularly regarding genomic stability, toxin production, and host–microbe interactions. Addressing these uncertainties requires systematic studies that integrate genomic analyses, toxicological assays, and well‐designed clinical trials to establish reliable safety profiles. At the same time, excessively conservative or prolonged approval processes at the research and early development stages may postpone the evaluation and eventual use of promising strains, particularly those derived from the human gut microbiota that could deliver tangible health benefits. Safety frameworks should therefore aim for a balance between rigorous risk assessment and timely access to innovation.

Another critical challenge is the absence of globally harmonized safety guidelines. Although EFSA, FDA, and other national authorities have issued individual frameworks, discrepancies in cut‐off values, test requirements, and data interpretation create inconsistencies in regulatory decisions (Marco et al. 2021; Sanders et al. 2019). Such heterogeneity increases the economic and temporal burden of probiotic development, as companies must adapt to multiple regulatory systems. This fragmented environment may further encourage firms to focus on repeatedly launching only well‐established strains while slowing or avoiding investment in novel candidates. As a result, strains with genuine health benefits may reach consumers much later than they should. Internationally coordinated standards would streamline safety assessments, reduce duplication of testing, promote innovation, and facilitate the global commercialization of probiotic products while strengthening consumer confidence.

Ultimately, safety evaluation serves as both the foundation and the gatekeeper of probiotic development. Early stage assessments, such as antibiotic resistance and hemolysis testing, remain indispensable for ensuring baseline safety (Rychen et al. 2018), while emerging challenges surrounding NGPs and fragmented international guidelines highlight the need for more comprehensive and harmonized approaches. Taken together, these regulatory safety requirements not only determine which strains can progress but also shape the overall trajectory, cost, and feasibility of probiotic innovation.

### 2.2 Functional Discovery

During the screening phase for evaluating the functional potential of probiotic strains, commonly assessed indicators include adhesion to the intestinal mucosa, antimicrobial activity, and immunomodulatory capacity. These properties serve as key predictors of a strain's ability to survive the harsh conditions of the upper gastrointestinal tract, reach the intestine, and exert beneficial physiological effects. Adhesion to the intestinal epithelium is a critical factor for colonization and interaction with the host microbiota. Antimicrobial activity, often mediated by competitive exclusion or metabolite production, reflects the strain's ability to suppress pathogens. Likewise, immunomodulatory properties indicate a potential positive influence on the host immune system. For example, *Lacticaseibacillus (Lcb*.) *rhamnosus* GG (LGG), known for its strong adhesion capacity, contributes to the stability of the intestinal microbiome (Deepika et al. 2009; Shi et al. 2020).

Several *Lactobacillus* strains have demonstrated strong inhibitory effects against pathogens, including *Vibrio parahaemolyticus*, *Listeria monocytogenes*, and *Shigella sonnei* (Shokryazdan et al. 2014). In addition, many strains influence innate and adaptive immune responses, such as modulation of Th1/Th2 balance and anti‐inflammatory activity, thereby contributing to the prevention of immune‐related disease (Kowalczyk et al. 2024).

Beyond these classical traits, emerging applications are being explored, such as probiotic interventions in dry eye disease (Tavakoli et al. 2022) and female urinary tract infections (Qasemi et al. 2023). Other novel bioactivities, such as the reduction of hyperuricemia (Zhao et al. 2022) and detoxification of uremic compounds (Chen et al. 2022), are also gaining attention.

However, even strains that exhibit strong functional potential may not be commercially viable if their viability declines drastically during processing and storage. For instance, a strain with excellent adhesion or antimicrobial activity may fail to survive freeze‐drying or high‐temperature storage, making it unsuitable for product development. Thus, functional screening must be considered alongside industrial robustness parameters.

Although in vitro assays remain indispensable for rapid and cost‐effective screening, their translational validity is limited. Acid and bile tolerance, adhesion to epithelial cells, or bile salt hydrolase (BSH) activity are reliable indicators of physiological robustness, yet these measures frequently fail to predict consistent clinical efficacy. For instance, LGG and *Bifidobacterium animalis* BB‐12 show strong concordance between in vitro gastrointestinal survival traits and human outcomes (Capurso 2019; Jungersen et al. 2014), whereas other strains with comparable in vitro performance have not shown reproducible benefits in gastrointestinal disorders (Quigley 2022). Similarly, in vivo studies provide important mechanistic insights, but interspecies differences in gut physiology and immune responses limit their predictive power for human efficacy (Quigley 2022).

To illustrate this translational gap more clearly, Table 1 summarizes representative examples of probiotic strains, comparing in vitro assays, in vivo outcomes, and clinical evidence side by side. This comparative overview highlights both the strengths and limitations of discovery methods and emphasizes the need for careful interpretation of screening data. A more detailed dataset is provided in Table S2 for reference. The emerging NGPs (e.g., *Akkermansia muciniphila*, *Anaerobutyricum soehngenii*) further highlight this translational gap. Although preclinical models robustly demonstrate metabolic and anti‐inflammatory effects, human evidence remains preliminary, often restricted to small pilot trials (Depommier et al. 2019; Gilijamse et al. 2020). Some promising strains, such as *Parabacteroides goldsteinii*, have accumulated substantial evidence from animal studies (Lai, Lin, Huang, et al. 2022; Lai, Lin, Chen, et al. 2022). However, interventional human trials have not yet been reported in the published literature. This discrepancy underlines a key challenge: functional discovery should be contextualized as an early gate in probiotic development, whereas long‐term stability, safety, and reproducibility ultimately determine commercial viability.

**TABLE 1:** Comparative overview of probiotic functional discovery and commercialization.

Strain (trademark)	Type and origin	In vitro evidence	In vivo evidence	Clinical evidence	Commercialization status	Product category	Patent	References
*Lcb. rhamnosus* GG (LGG)	Conventional, healthy human feces	Acid/Bile tolerance; adhesion to enterocytes; antimicrobial activity	Preclinical evidence supports intestinal mucosa protection and pathogen inhibition	Multiple RCTs: reduction of acute diarrhea, alleviation of IBS/IBD symptoms, immune modulation	Globally established; DSHEA; QPS	Food (restricted in the United States); supplement	Expired patent on strain; patent on indication and composition	Capurso (2019), Steele (2022), National Institutes of Health (NIH) (2021), EFSA Biohaz Panel (2025)
*Bifidobacterium animalis* subsp. lactis BB‐12 (BB‐12)	Conventional, dairy cultures	Acid/Bile tolerance; BSH activity; mucus adhesion	Improved gut barrier, reduced diarrhea in animal models	Multiple RCTs: improved bowel function, prevention of diarrhea, reduced respiratory infections	Globally established; GRAS; QPS	Food; supplements	Expired patent on strain; patent on indication and composition	Isolauri et al. (), Jae Ho et al. (2016), Jungersen et al. (2014), Salminen (2019), Wong et al. (2019), FDA (2019), EFSA Biohaz Panel (2025), Nordström et al. (2021), FDA (2017), EFSA Biohaz Panel (2025)
*Lpb. plantarum* 299v (LP299V)	Conventional, healthy human intestinal mucosa	GI survival; epithelial adhesion; antimicrobial safety profile	Gut colonization, modulation of local immune markers	RCTs: IBS symptom alleviation, enhanced iron absorption	Widely commercialized; GRAS; QPS	Food; supplements	Expired patent on strain; patent on indication	FDA (2025), Kaźmierczak‐Siedlecka et al. (2020), EFSA Biohaz Panel (2025)
*Akkermansia muciniphila*	NGP, human gut	Mucin degradation/utilization; outer membrane protein activity	Improved insulin sensitivity, lipid lowering, reduced inflammation in obesity/NAFLD/T2D models	Clinical pilot trials: improved insulin sensitivity and cholesterol profile	Pilot commercialization; NDI (live cell); novel food (pasteurized)	Supplement	Patent on strain level; patent on species level covering processing and claimed indications	He et al. (2021), Langella et al. (2021), Seo et al. (2020), U.S. National Library of Medicine ()

Abbreviations: GRAS, generally recognized as safe; IBD, inflammatory bowel disease; IBS, irritable bowel syndrome; NDI, new dietary ingredient; NGP, next‐generation probiotics; QPS, qualified presumption of safety.

As illustrated in Table 1, conventional probiotics with extensive validation, such as LGG and BB‐12, have successfully translated from in vitro promise to clinical and commercial application, whereas next‐generation candidates like *A. muciniphila* remain at an early stage with limited human data. This comparison reinforces that functional screening alone cannot guarantee translational or commercial success.

Therefore, functional screening should be interpreted as a hypothesis‐generating step rather than a definitive predictor of marketable efficacy. A critical balance between in vitro discovery, in vivo validation, and well‐controlled human trials is essential to bridge the translational gap and to avoid overestimating strains that may fail at later stages of development.

### 2.3 Stability Assessments

For industrialized probiotics, freeze‐drying is considered an essential process for long‐term preservation and product development. However, because the survival rate of probiotic strains during freeze‐drying is highly influenced by multiple factors, quantitative evaluation and process optimization are crucial. First, the pre‐freezing treatment conditions significantly affect cell viability. Extremely fast or slow cooling rates can influence intracellular and extracellular water migration and ice crystal formation, potentially damaging the cell membrane. Therefore, setting an optimal cooling rate and freezing temperature is essential (Abdelhady et al. 2024). Second, the use of cryoprotectants is a key strategy for minimizing cellular damage during the freeze‐drying process and improving survival rates. Additives, such as skim milk powder, trehalose, and sodium ascorbate, have been reported to form protective barriers around microorganisms, thereby reducing freezing damage (Jalali et al. 2012). Third, water activity is directly related to product stability. When water activity is maintained below 0.25, microbial metabolism is suppressed and favors the maintenance of viable cell counts during storage. In contrast, at water activities exceeding 0.33, survival rates may decrease significantly (Kurtmann et al. 2009; Poddar et al. 2014). Fourth, the storage conditions after freeze‐drying also play a critical role in maintaining probiotic viability. In general, low‐temperature storage, such as refrigeration at 4°C, is effective for preserving viability, whereas high‐temperature and high‐humidity environments can cause rapid declines in viable cell counts (Abe et al. 2009). Probiotic commercialization requires comprehensive consideration of these factors in the design of freeze‐drying conditions and stability evaluations.

Although freeze‐drying has been extensively investigated as a preservation method for probiotics, most published studies have been carried out under laboratory‐scale conditions rather than full industrial settings. Recent reviews have synthesized these findings but still emphasize encapsulation, protective agents, and pretreatment strategies primarily validated in research laboratories, with limited reference to industrial‐scale validations (Agriopoulou et al. 2023; Ge et al. 2024). One key consideration is that this imbalance highlights a critical gap: Although industrial practices undoubtedly apply preservation know‐how, peer‐reviewed literature has not sufficiently captured or critically analyzed these commercial cases. As a result, academic insights may not fully reflect the actual challenges and solutions encountered in manufacturing environments. Moreover, companies often treat process conditions, long‐term stability data, and packaging innovations as trade secrets because such information directly underpins their competitive advantage. This strategic nondisclosure creates an additional barrier that limits the availability of critical evidence in scientific literature. Bridging this gap will require not only laboratory optimization but also collaborative studies with industry that report quantitative survival data, long‐term stability under supply‐chain conditions, and the influence of packaging and humidity control. Such integration would provide a more realistic foundation for designing robust stability strategies and accelerate the successful commercialization of NGPs.

The commercialization of probiotics requires comprehensive consideration of these factors in the design of freeze‐drying conditions and stability evaluations. However, most discussions to date have remained focused on laboratory‐scale conditions, leaving academic understanding of long‐term preservation and quality maintenance under industrial settings limited. Although some reviews have briefly noted this limitation, few have examined in depth the structural reasons behind the scarcity of industrial data (Ge et al. 2024; Lin, Si, et al. 2024; Wang et Zhong 2024). In practice, companies often treat survival data, storage stability, and packaging strategies as proprietary information directly tied to competitive advantage, which creates a barrier to their dissemination in peer‐reviewed literature. This lack of transparency not only constrains academic insights but also hampers the development of harmonized stability standards essential for regulatory approval and consumer trust. Therefore, future research should go beyond simply calling for more industrial‐scale validations and instead focus on overcoming the current disjunction between academic research and industrial practice, for example, through collaborative studies, data‐sharing incentives, and regulatory frameworks that promote transparency.

### 2.4 Strategies for the Isolation of Lead Probiotics

In the probiotics industry, translating high‐potential strains newly identified from academic research into widely accessible consumer products faces numerous challenges. In practice, even strains with excellent functional properties may be unsuitable for commercialization if they exhibit low survival rates under the harsh conditions used during manufacturing processes, which often involve exposure to heat, moisture, or oxygen, or during storage when temperature and humidity conditions may vary (Abe et al. 2009). Strains with inherently high stability can maintain high viable cell counts during drying, even with minimal use of cryoprotectants. However, when maximizing the industrial profitability of a less stable strain is a goal, processes that can increase post‐drying survival rates and long‐term storage stability become essential. As a result, industrial producers often face a dilemma when prioritizing the functional efficacy and stability of candidate probiotic strains.

**FIGURE 3:**
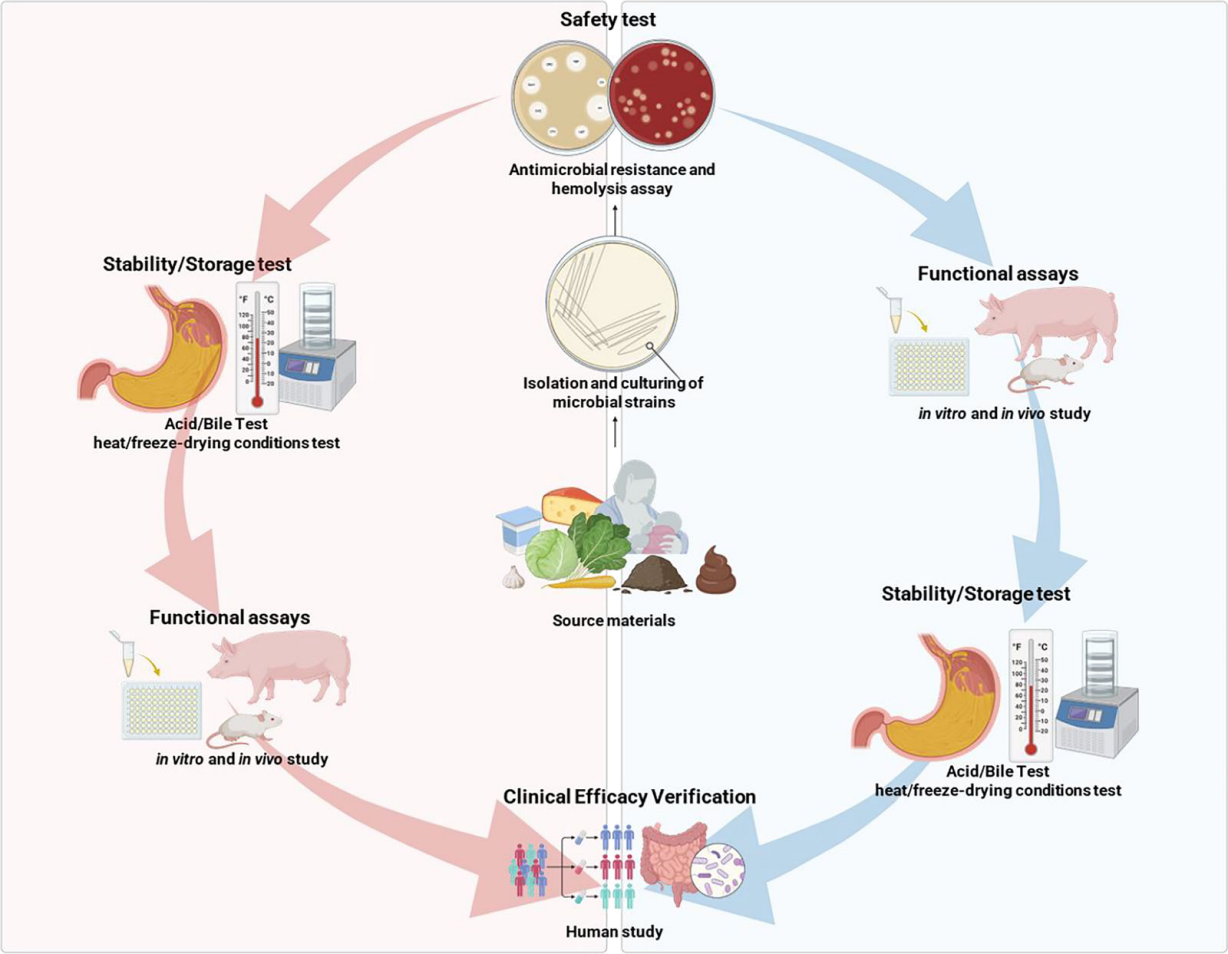
Dual‐screening strategies for selection probiotic strains with enhanced safety, stability, and functionality.

This challenge can be met by implementing dual screening strategies, as these can expand the pool of viable strains for consumer applications. As illustrated in Figure 3, a dual screening strategy proceeds along two parallel evaluation tracks following strain isolation. In one track, strains under consideration undergo safety assessments, including tests for antibiotic resistance and hemolytic activity, followed by viability evaluations under stress conditions, such as exposure to acid, bile, heat, and freeze‐drying. In the second track, the functional properties, such as antimicrobial activity, immunomodulatory effects, and metabolite production, are then assessed. This approach (Figure 3) is widely used due to its clear structure and high efficiency. However, a major limitation is the potential exclusion of strains that possess valuable functional traits but exhibit poor initial stability.

Alternatively, strains can be first evaluated for safety and then screened for functional potential. As safety is the most critical prerequisite for probiotic development, this order of prioritization is logical. Functionally potent strains retained through this approach, even if initially unstable, can subsequently undergo stabilization processes (Figure 3), such as encapsulation, protective agent addition, or stress‐adaptation techniques (e.g., cross‐protection), thereby enabling their health‐promoting effects to become available to consumers. Although this approach often involves longer development timelines and higher production costs, it allows the inclusion of high‐value strains that would otherwise be discarded. Therefore, it highlights the importance of developing more efficient and cost‐effective stabilization methods to broaden the range of strains available for industrial use (Figure 3).

Enhancing the stability of functionally potent strains offers significant advantages, both industrially and from the consumer's perspective. Consumers benefit by gaining access to highly functional strains at a lower cost, whereas producers can increase their market competitiveness by developing high‐performance strains that meet a broader range of health indications. Consequently, any adopted dual‐screening strategy must simultaneously consider both functionality and stability at the early stages of strain selection. This type of approach will go beyond simple screening and enable the early identification of strains with true industrial potential.

Beyond screening approaches, other microbiological strategies have been proposed to overcome stability challenges during probiotic production and storage. One such method involves repeated exposure of strains to nonlethal levels of stresses, such as high temperature or acidic conditions, which can induce cross‐protection and enhance stress resistance (Gueimonde and Sanchez 2012). However, for these induction‐based strategies to become truly industrially applicable, multiple dimensions must be systematically evaluated.

First, feasibility depends not only on whether stress induction is successful under laboratory conditions but also on the reproducibility and stability of the induced effect. For example, short‐term heat stress can trigger heat‐shock protein expression in certain LAB, improving survival during subsequent drying. However, this effect varies significantly across strains and even between different batches of the same strain, creating challenges for process standardization (Abe et al. 2009).

Second, scalability represents a key bottleneck for industrial adoption. Induction methods effective at shake‐flask or pilot scales may not retain uniformity and reproducibility in large‐scale fermenters or continuous drying processes. For instance, osmotic or acid induction may be relatively easy to implement in small batches but difficult to control precisely in large‐scale operations, potentially prolonging fermentation cycles and reducing yield (Saarela et al. 2009).

Third, cost efficiency is a decisive factor. Although induction alone may appear inexpensive because it does not require costly additives, hidden costs, such as increased energy consumption, extended process times, and additional quality control, must be considered. We therefore propose a hybrid strategy that combines mild induction with low‐dose protective agents to balance cost and efficacy (Gueimonde and Sánchez 2012).

Finally, regulatory acceptance remains decisive for the translation of induction‐based traits into market‐ready products. Current frameworks such as the FDA's GRAS system and EFSA's QPS process require demonstration of genomic stability, absence of transferable antibiotic resistance, and safety validation of newly induced traits (Sanders et al. 2010). Although transient phenotypic adaptations are generally acceptable, stable genetic modifications may be categorized as GMOs, raising additional regulatory hurdles (Garg et al. 2024; Koutsoumanis et al. 2020; Wright 2005).

Taken together, research on induction‐based stabilization should not be limited to demonstrating improved survival rates. Instead, it must be systematically evaluated along the full chain of mechanistic understanding, process scale‐up, cost‐effectiveness, and regulatory compliance. Only with such an integrated approach can induction strategies be successfully translated from laboratory findings into competitive industrial technologies.

In addition, real‐world case analyses of academic‐industry collaborations provide clear evidence of how laboratory discoveries are translated into consumer‐ready probiotic products. A representative example is LGG, which was originally isolated and patented by researchers at Tufts University. The strain was subsequently licensed to Valio for early commercialization and later disseminated globally by Chr. Hansen. This represents a classic case of an academic discovery successfully translated into industrial applications, supported by extensive clinical validation (Capurso 2019).

By contrast, *Limosilactobacillus (Lm.) reuteri* DSM 17938 and *B. animalis* subsp. *lactis* BB‐12 illustrate the reverse model, in which industry‐developed strains gained scientific credibility through academic collaborations. DSM 17938 was derived by BioGaia through plasmid curing of the parent strain ATCC 55730 to remove transferable antibiotic resistance, after which its safety and efficacy were confirmed through multiple randomized controlled trials and FDA‐IND regulated studies in collaboration with academic centers (Rosander et al. 2008; Savino et al. 2010). Similarly, BB‐12, developed by Chr. Hansen, has been extensively validated by independent academic and clinical research groups, with evidence supporting its safety and efficacy in both children and adults (Jungersen et al. 2014; Taipale et al. 2012).

Taken together, these cases highlight two complementary pathways of translational investigation: academic‐to‐industry translation (LGG) and industry‐to‐academic validation (DSM 17938 and BB‐12). These cases illustrate that successful translation of probiotics requires not only strain‐level innovations but also strategic partnerships that integrate academic discovery with industrial validation.

## 3 Environmental Stress and Screening Strategies for Identifying Robust Probiotic Strains

**FIGURE 4:**
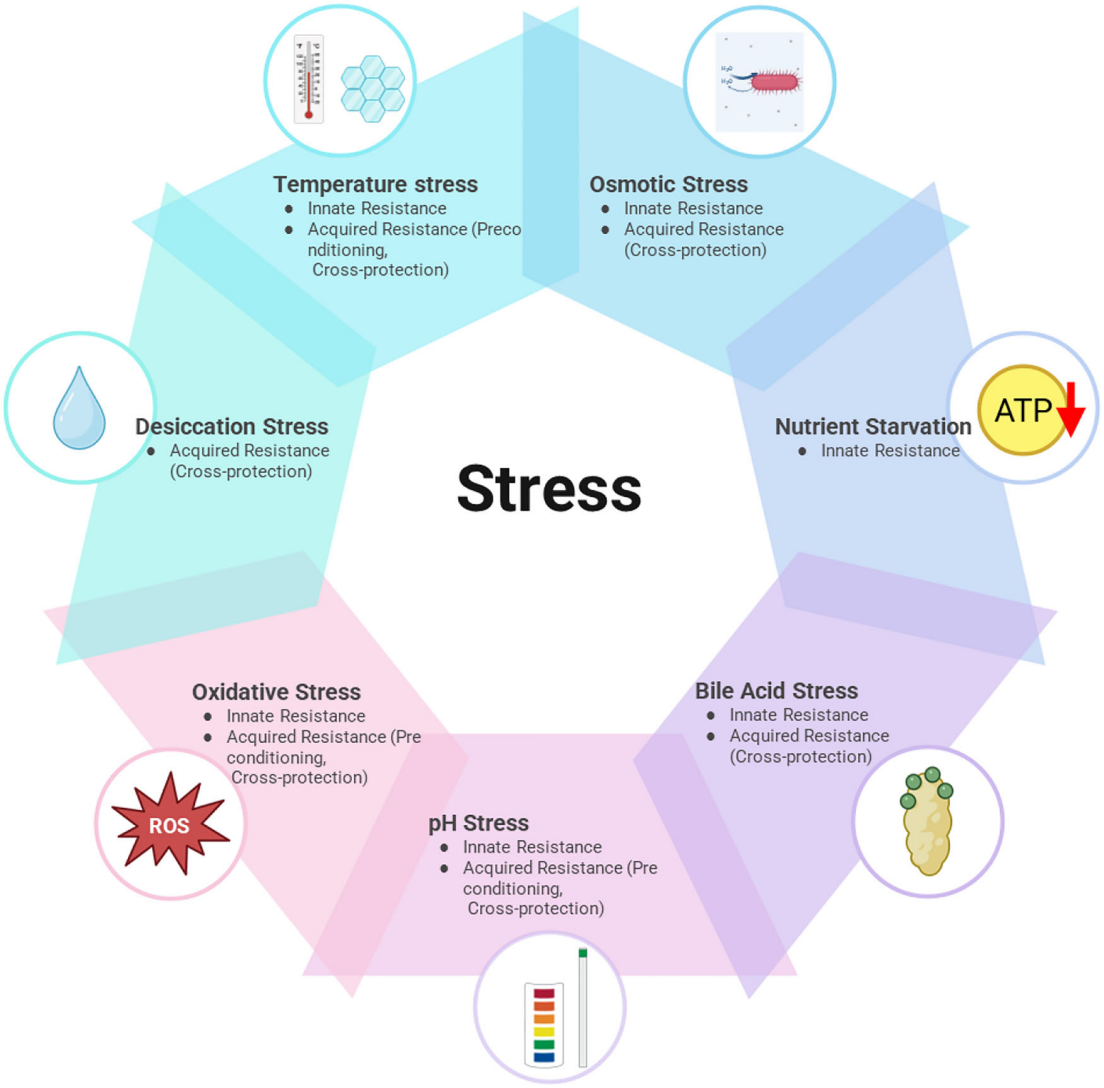
Stress factors and adaptive strategies influencing probiotic viability from production to gut colonization.

Probiotics are exposed to various environmental stressors throughout production, storage, and consumption processes, all of which can critically affect their viability and shelf life. However, true stability should go beyond mere shelf life to encompass more practical indicators, such as the numbers of viable cells that not only survive within capsules or beverages but also beneficially colonize the gut after ingestion. Key stress factors that limit viability include temperature, osmotic pressure, oxidative stress, acidity, bile salts, water activity, and nutrient deficiency (Figure 4), and these factors exert diverse influences across the entire production and storage chain (Ayyash et al. 2021; Sionek et al. 2024). Furthermore, these stressors often act synergistically, highlighting the importance of selecting strains with multiple stress resistances.

**FIGURE 5:**
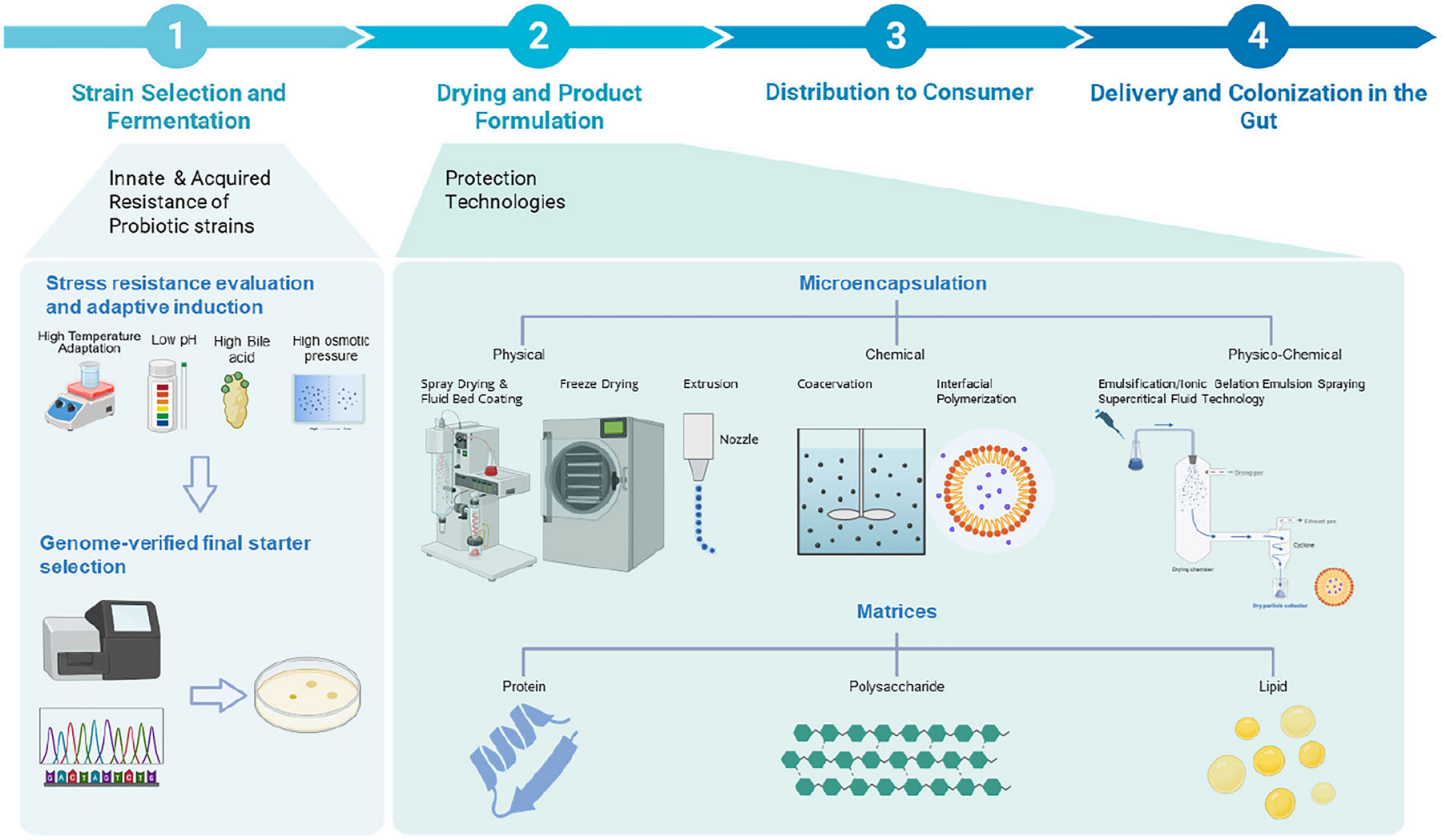
Strategies for improving probiotic stability from manufacture to gut delivery.

Accordingly, screening strategies that mimic these environmental challenges can serve as effective approaches for identifying lead probiotic strains (Figure 5). As illustrated in Figure 5, the entire pipeline of probiotic development spans from the initial isolation and screening of candidate strains to large‐scale production, formulation, and eventual consumer delivery. The present section focuses on the first stage of strain selection, in which the identification or induction of stress‐resistant candidates is crucial. Strains that demonstrate innate or acquired tolerance to harsh conditions such as heat, acidity, bile, and desiccation are more likely to withstand industrial processing and storage, thereby providing a competitive advantage in the marketplace. By integrating stress‐resistance screening into early discovery, companies can reduce downstream losses, accelerate commercialization, and strengthen their position in the probiotic industry. These strategies can include screening strains based on their innate stress resistance, as shown in Table 2 and Figure 4. In Table 2, it should be noted that not all studies reported exact survival percentages or CFU/mL values, and in some cases, only qualitative outcomes were available. This inconsistency limits the possibility of direct numerical comparison across studies and highlights the need for cautious interpretation and context‐specific application of the available data. Stress tolerance can generally be categorized into innate resistance, which reflects genetic and physiological traits inherent to the strain, and acquired resistance, which arises from adaptive responses induced by prior exposure to stress. Recognizing this distinction provides a basis for developing strategies aimed at strengthening weaker traits and guiding the selection of candidate microorganisms with superior robustness. Systematic identification and characterization of these resistance features are therefore essential for advancing the development of functional probiotics that can withstand industrial and physiological challenges. The induction of tolerance to key environmental stressors, such as acidity, bile, and heat, facilitates the early identification of strains with high industrial viability and consumer applicability.

**TABLE 2:** Intrinsic stress tolerance of selected probiotic strains and corresponding evaluation methods.

Type of stress	Evaluation methods	Strains survived	References
Heat stress	45°C for 30 min	*Lpb. plantarum* Lp998	Ferrando et al. (2015)
	65°C for 10 min, cultivitable at 65°C	*Lcb. casei* ATCC393	Haddaji et al. (2015)
Osmotic stress	8% NaCl for 24 h	*Lpb. plantarum* D31	Yao, Xie, et al. (2020)
	1%–4% NaCl for 18 h	*Lcb. casei* IMAU60214	Rocha‐Ramírez et al. (2021)
Oxidative stress	2 mM H_2_O_2_ for 30 min	*Lcb. casei* CI4368	Zotta et al. (2014)
	5 mM H_2_O_2_ for 30 and 60 min	*Lpb. plantarum* CAUH2	Zhai et al. (2020)
Acid stress	pH 2.0 for 3 h	*Lcb. casei* Zhang	Wang et al. (2009)
Bile salt stress	0.2% oxgall for 2 h	LGG	Koskenniemi et al. (2011)
	3.6% bovine bile, 24 h	*Lpb. plantarum* IMC510	Prete et al. (2021)
Nutritional deficiency	Transferred to fresh medium (without glucose) for up to 2 years	*Lactococcus lactis* subsp. *lactis* LL41‐1	Kim et al. (2001)

Strategies that explore stabilization technologies, particularly microencapsulation, have also been widely applied. These technologies, especially during drying and storage, can enhance resistance to external stresses to improve probiotic survival, support industrial scalability, and strengthen consumer confidence (Yao, Xie, et al. 2020).

### 3.1 Innate Resistance to Environmental Stressors

#### 3.1.1 Temperature Stress

Probiotics generally maintain optimal growth and metabolic activity between 30°C and 40°C, corresponding to the physiological range of mesophilic microorganisms, such as *Lactobacillus* spp. However, during industrial production, storage, and consumption, probiotic strains are often exposed to atypical temperature fluctuations, which can significantly impair viability. Heat stress can reduce membrane fluidity and denature proteins, with adverse effects on permeability and enzyme activity, whereas cold stress may cause physical damage by rigidifying membranes, destabilizing nucleic acids, and promoting ice crystal formation (Bustos et al. 2025). Fortunately, some strains exhibit thermal adaptability. For example, *Lpb*. *plantarum* strains (Lp 813 and Lp 998) grow at 45°C for over 24 h, and Lp 813 and Lp 998 remained cultivable following brief exposure to 55°C (Ferrando et al. 2015), although detailed survival rates were not provided. Notably, reports for *Lacticaseibacillus* (*Lcb*.) *casei* ATCC 393 at 65°C indicate short‐term survival under heat exposure rather than sustained growth (Haddaji et al. 2015). As summarized in Table 2, reported thermotolerance endpoints range from sustained growth at 45°C to brief cultivability at 65°C; because growth, survival, and cultivability capture distinct phenotypes, cross‐study numeric comparisons should be interpreted with caution. These heat‐resistance traits are valuable for industrial processes like spray‐drying or heat pretreatment, where probiotics may face temperatures above 50°C.

The identification of robust strains typically involves screening protocols that expose candidate strains to high or low temperatures and assess survival, growth, and resilience (Gueimonde and Sanchez 2012). Stepwise models starting with severe conditions are often used to mimic industrial stress. Protective strategies, such as coating cells with agents like trehalose, sucrose, whey protein, or casein, are also used to enhance thermal tolerance. These compounds, which help stabilize membranes and increase the glass transition temperature, can improve survival during freeze‐drying or thermal processing (Gueimonde and Sanchez 2012; Sionek et al. 2024).

#### 3.1.2 Osmotic Stress

Like most microorganisms, probiotics exhibit optimal viability when their intracellular environment is maintained under balanced osmotic conditions. Excessive external osmotic pressure disrupts water equilibrium, causing membrane shrinkage, protein denaturation, and enzyme inactivation, which together impair cellular function (Burg et al. 2007). High concentrations of salt or sugars, as well as dehydration, can induce osmotic stress by drawing water out of the cell, with consequent ion imbalances and reduced protein stability that ultimately decrease probiotic viability. Most probiotic strains can tolerate NaCl concentrations of 1%–2%, but growth inhibition and increased cell death are commonly observed at salinities of 3%–4% or higher (Rocha‐Ramírez et al. 2021). Therefore, controlling osmotic pressure during cultivation is essential for maintaining both production efficiency and cell viability. This can be achieved by adjusting NaCl or sugar concentrations in the growth medium or by adding osmoprotectants, such as trehalose, glutamine, or glycine betaine. Similar protective strategies can also be applied during freeze‐drying, as these agents can help mitigate freezing‐related moisture loss and cellular damage (Le Marrec 2011).

Interestingly, strains that survive high NaCl conditions often show enhanced resistance to dehydration stress, including dehydration induced by freeze‐drying. *Lpb. plantarum* D31 demonstrated growth under 8% NaCl, as indicated by an increase in OD_600nm_ values from approximately 0.5 to nearly 1.0, whereas the comparator strain exhibited no detectable growth. In addition, D31 showed excellent survival following freeze‐drying, suggesting a potential link between osmotic adaptability and dehydration tolerance (Yao, Yang, et al. 2020). As in Table 2, D31's performance under 8% NaCl was assessed by OD_600nm_ increase, whereas other studies report CFU‐based survival; aligning endpoints within a screening workflow is therefore advisable. For industrial applications, a two‐step screening strategy is recommended in which a test strain's survival under high‐salt conditions is first tested, followed by viability assessments before and after freeze‐drying. This dual‐screening method provides a practical approach for selecting robust strains with superior stability and shelf life under real‐world processing conditions.

#### 3.1.3 Desiccation Stress

Water activity (*a*_w_), as one of the key physical factors that critically affect the survival, growth, and metabolic activity of microorganisms, is regarded as a core indicator in the stability assessment of probiotics. In general, most microorganisms exhibit active growth at *a*_w_ values above 0.90, whereas growth and metabolism are significantly inhibited under conditions where *a*_w_ falls below 0.60 (Stevenson et al. 2015). In powdered or dried probiotic products, *a*_w_ is often below 0.60, imposing severe desiccation stress that can damage membrane and protein structures and destabilize enzymes (Broeckx et al. 2016). Freeze‐drying, a commonly applied stabilization method for probiotics, imposes both mechanical and dehydration stresses. For instance, in the case of *Bifidobacterium longum*, survival rates after freeze‐drying increased from 28% to 49% when optimal protectants and proper shelf temperatures were applied (Haindl et al. 2020). However, in less optimal storage or higher temperature conditions, survival drops significantly. Residual moisture content and storage temperature substantially affect viability losses; *B. longum* cells stored at +20°C without protectants lose far more viability than those stored at +4°C with protectants (Haindl et al. 2020). Protective agents, such as trehalose, lactose, sucrose, milk proteins, and glycerol, can help stabilize cells by replacing water molecules and preserving membrane integrity. Trehalose promotes vitrification, which enables rapid recovery upon rehydration (Wendel 2022; Broeckx et al. 2016; Mendonça et al. 2022). Details on protective excipients are consolidated here; other sections refer to this discussion to avoid redundancy.

Storage stability is equally critical, as dried probiotics are sensitive to humidity and temperature and benefit from moisture‐barrier packaging and refrigeration. For example, high humidity can cause uneven rehydration and membrane rupture. Notably, the effects of desiccation and osmotic stress share a common mechanism: the promotion of imbalances in intracellular water. Both lead to membrane shrinkage, protein aggregation, and metabolic inactivation. However, due to this overlap, screening for desiccation resistance may also reflect a strain's osmotic stress tolerance.

#### 3.1.4 Oxidative Stress

Probiotics are typically facultative anaerobes or microaerophiles that show optimal viability under low‐oxygen conditions. Thus, oxygen exposure during production, storage, or consumption can significantly reduce their survival. The generation of reactive oxygen species (ROS) due to oxygen exposure can cause membrane lipid peroxidation, protein denaturation, and DNA damage (Feng and Wang 2020). Unlike aerobic bacteria, most probiotics lack sufficient levels of ROS‐scavenging enzymes, such as catalase and superoxide dismutase (SOD), making them more vulnerable to oxidative stress. During storage, oxygen and moisture infiltration into powdered or freeze‐dried formulations can accelerate ROS accumulation and lead to cell death. To mitigate this, antioxidant compounds, such as ascorbic acid and tocopherol, are often added to formulations to improve their post‐freeze‐drying viability (Rodklongtan et al. 2022). Packaging technologies can also play a crucial role, with oxygen absorbers and nitrogen or carbon dioxide flushing widely used to minimize oxygen levels and extend probiotic shelf life.

Some strains, such as *Lpb. plantarum* CAUH2, exhibit adaptive responses to oxidative stress by upregulating enzymes like SOD under H_2_O_2_ exposure. CAUH2 showed negligible reduction in cell counts after 30 or 60 min of exposure to 5 mM H_2_O_2_ and exhibited only a slight decrease of approximately 0.3 log CFU/mL at 7 mM, supporting its strong resistance to oxygen‐rich environments (Zhai et al. 2020). Consistently, Zotta et al. (2014) reported that, among 184 tested strains, four *Lcb. casei* isolates (CI4368, N87, N811, and N2014) unexpectedly displayed catalase‐like activity and were able to survive exposure to 2 mM H_2_O_2_ (Zotta et al. 2014). These strains can be screened by evaluating their survival under oxidative stress, making them promising candidates for the development of oxygen‐resistant probiotics.

#### 3.1.5 pH and Bile Salt Stress

Probiotics, due to their ability to produce organic acids, generally survive well in mildly acidic environments (pH 5.0–6.5). However, strongly acidic conditions below pH 4.5 pose a challenge for survival in gastric environments, particularly for *Bifidobacterium* species, as the pH ranges from 2.0 to 3.0 (Takahashi et al. 2004). Acidic stress can denature enzymes, alter membrane permeability, and overload proton pumps, ultimately leading to cell death and emphasizing the importance of selecting acid‐resistant strains. For instance, freshly prepared cultures of *Lcb. casei* Zhang demonstrated survival rates of 31% and 69% after 3 h of exposure at pH 2.0 and 2.5, respectively, highlighting its relatively strong acid tolerance compared with many other strains (Wang et al. 2009). As summarized in Table 2, acid tolerance endpoints vary in intensity and duration, underscoring the need to standardize exposure protocols for comparative screening.

In contrast, the small intestine presents a neutral to alkaline environment (pH 7–8) due to the presence of bile salts. Although most probiotics adapt well to acidic conditions, their adaptability to alkaline stress is limited. Alkalinity can impair enzymes and disturb metabolic balance, although its impact is generally milder than that of acid stress. Nevertheless, alkalinity resistance remains a key criterion in strain selection. Bile salts, though essential for lipid digestion, disrupt cell membranes and reduce probiotic viability (Ruiz et al. 2013), and probiotic bile tolerance is strain specific. For example, LGG maintains viability under 0.2% oxgall bile, whereas *Lpb. plantarum* IMC510 survived in 3.6% bovine bile for 24 h, showing no statistically significant difference in viable counts compared with the untreated control (Prete et al. 2020; Koskenniemi et al. 2011). Because oxgall and bovine bile differ in composition and critical micelle concentrations, bile‐tolerance outcomes are matrix dependent, limiting direct numeric comparisons across studies (Table 2). Thus, evaluating both acid and bile resistance is crucial. Recently developed gastrointestinal simulators now allow precise assessment of strain survival through the gastrointestinal tract, thereby aiding effective probiotic screening (Ayyash et al. 2021).

#### 3.1.6 Nutrient Starvation

During storage, probiotics may experience nutrient limitation depending on the composition of the surrounding matrix (Sionek et al. 2024). In freeze‐dried form, they remain dormant with minimal metabolism. Although this state preserves initial viability, prolonged nutrient deprivation eventually leads to intracellular metabolite depletion, waste accumulation, and loss of vitality. Survival during storage relies on the presence of internal energy reserves, which in some bacteria include glycogen or polyphosphate, but over time viability decreases due to ATP depletion, membrane damage, and protein dysfunction. LAB generally lose viability during storage, and the extent of this decline varies depending on environmental factors such as temperature, residual moisture, and oxygen. Long‐term nutrient deprivation can also induce survival responses; for instance, *Lactococcus lactis* survived for up to 350 days under glucose starvation, although viable counts gradually decreased, and in some conditions, no cells were detectable after approximately 110 days (Kim et al. 2001).

Ingested probiotics, especially those delivered in encapsulated forms without buffers or nutrients, also experience stress from acid, bile, and osmotic pressures in the absence of external energy sources. They depend on their endogenous reserves and experience metabolic arrest and cell death when those reserves are exhausted. To improve resilience, preconditioning methods, such as cultivation under nutrient‐limited conditions, have been explored. These methods induce adaptive changes, including storage polysaccharide accumulation and membrane protein expression, that enhance survival during drying and gastric stress (Mills et al. 2011). However, excessive nutrient limitation may impair repair capacity and enzymatic activity, so this strategy requires cautious application.

Protective agents, such as trehalose, lactose, and whey protein, are known to stabilize cell structures during freeze‐drying. Encapsulation systems using alginate, cellulose, or resistant starch help maintain dormant stability during storage (Santivarangkna et al. 2011). Combinations of preconditioning, protective matrices, and optimized packaging are essential to reduce vitality losses and preserve probiotic functionality during intestinal delivery.

### 3.2 Acquired Stress Resistance Strategies in Probiotics: Preconditioning and Cross‐Protection

The commercial success of probiotic products largely depends on the selection of strains capable of withstanding the various environmental stresses, such as heat, acidity, bile, and osmotic pressure, that are encountered during their production, storage, and consumption of a given probiotic. Recently, strategies aimed at inducing acquired resistance to these stresses have been actively explored. This section provides an academic review of representative approaches for enhancing stress tolerance in probiotics and discusses relevant case studies (Table 3).

**TABLE 3:** Strategies for inducing acquired stress tolerance in probiotic strains.

Stress preconditioning				
Stress challenge	Strain	Precondition	Observed effect	References
Heat (60°C, 1 h)	*Lpb. plantarum* KLDS 1.0628	Heat shock at 45°C for 1 h	Survival rate increased 31.38‐fold	Ma et al. (2021)
Heat (44°C, 10 min)	*Lpb. plantarum* LIP‐1	Heat treatment at 75°C for 40 s	The reduction of survival rate decreased from 1.05 to 0.36 log CFU/mL	Zhang et al. (2023)
Acid (pH 3.5, 2 h)	*Bifidobacterium longum* BBMN68 mutant	pH 4.5, 2 h	Survival rate increased 70‐fold (293 genes upregulated, 245 downregulated)	Jin et al. (2012)
Acid (pH 3.0, 6 h)	*Lactobacillus kefiranofaciens* M1	pH 5.0, 1 h	Survival rate increased 9.9‐fold	Chen et al. (2017)
Acid (pH 3.5, 1.5 h)	*B. longum*	150 cycles (pH 3.5, 37°C, 1.5 h)	Survival rate increased (289 genes downregulated, whereas 61 gene upregulated)	Wei et al. (2019)
Oxidative stress (H_2_O_2_ 3% (v/v))	*Lactobacillus johnsonii/gasseri*	Respiratory cultivation (O_2_, heme, menaquinone)	Enhanced oxidative stress resistance	Maresca et al. (2018)

Cross‐protection				
Stress challenge	Strain	Induction	Observed effect	References
Acid (pH 5.0, 30 min)	*Enterococcus faecium* HL7	Heat (60°C, 40 min)	Survival rate increased from 5.2% to 92.7%	Shin et al. (2019)
Ethanol (20%, 2 h)		Heat (52°C, 15 min)	Survival rate increased from 0.1% to 80.8%	
Acid (pH 3.0, 4 h)		Heat (52°C, 15 min)	Survival rate increased from 1.1% to 3.7%	
Alkali (pH 12, 4 h)		Heat (52°C, 15 min)	Survival rate increased from 0.3% to 3.0%	
Cold tolerance (−20°C, 14 days)	*L. plantarum* KLDS 1.0628	Acid (pH 4.0, 1 h)	Induced 10‐fold increases in cold tolerance	Ma et al. (2021)
H_2_O_2_ tolerance (5 mmol/L, 1 h)		Acid (pH 4.0, 1 h)	Induced 17‐fold increases in H_2_O_2_ tolerance	
Desiccation, Storage at 30°C, 14 weeks	*Lcb. rhamnosus* HN001	Heat shock (50°C, 30 min)	Control: 7.3‐log‐unit reduction; Induction: 1.6‐log‐unit reduction	Prasad et al. (2003)
Desiccation, Storage at 30°C, 14 weeks		Osmotically shock (0.6 M NaCl)	Control: 7.3‐log‐unit reduction; Induction: 2‐log‐unit reduction	
Heat tolerance (52°C, 2 h)	*Lactobacillus kefiranofaciens* M1	Bile salt (0.05%, 1 h)	Increased thermal tolerance 46.6‐fold	Chen et al. (2017)
Cold tolerance		Acid (pH 5.0, 1 h);	Induced 9.6‐fold increases in cold tolerance	
(−20°C, 12 days)				
Cold tolerance		Bile salt (0.05%, 1 h)	Induced 5.9‐fold increases in cold tolerance and	
(−20°C, 12 days)				

Stress preconditioning and adaptive laboratory evolution (ALE) are strategies designed to enhance microbial survival by repeatedly exposing probiotic strains to environmental stressors at sublethal levels. The aim of these approaches is to induce physiological and genetic adaptations that improve stress resistance. For instance, Ma et al. (2021) reported that *Lpb. plantarum* KLDS 1.0628 exhibited a 31.38‐fold improvement in survival after being subjected to a 1 h heat shock at 45°C, followed by lethal exposure at 60°C for 1 h (Ma et al. 2021). Similarly, Zhang et al. (2023) demonstrated that pretreating *Lpb. plantarum* LIP‐1 at 44°C for 10 min significantly reduced viability loss under subsequent heat exposure (75°C for 40 s), with log reduction values decreasing from 1.05 to 0.36 log CFU/mL (Zhang et al. 2023; Ma et al. 2021).

Jeon et al. (2021) also demonstrated the effectiveness of ALE‐based strategies by improving the heat resistance of *L. acidophilus* EG008. Through repeated exposure and selection, the strain's thermal tolerance increased from 65°C to 75°C. Whole‐genome sequencing revealed two single nucleotide polymorphisms (SNPs) associated with this enhanced resistance, suggesting underlying genetic adaptations that contributed to the acquired thermotolerance (Jeon et al. 2021). Jin et al. (2012) demonstrated that a 2 h acid exposure at pH 4.5 induced the development of an acid‐tolerant mutant of *B. longum* BBMN68, which showed a 70‐fold increase in survival when exposed to pH 3.5 conditions for 2 h. Transcriptomic analysis revealed extensive gene expression changes, including the upregulation and downregulation of hundreds of genes, indicating a complex regulatory response associated with the acquired acid resistance (Jin et al. 2012). Mechanistically, acid‐adapted derivatives can rewire envelope and metabolic traits relative to their parental strains; for example, altered fatty‐acid composition of the cell membrane at low pH, upregulation of aldehyde dehydrogenase and long‐chain acyl‐CoA ligase homologs, enhanced ammonium generation and transport, and remodeling of peptidoglycan biosynthesis collectively strengthen acid tolerance (Wei et al. 2019). Chen et al. (2017) reported a 9.9‐fold increase in survival of *Lactobacillus kefiranofaciens* M1 under acid challenge after preconditioning at pH 5.0 for 1 h. Additionally, for oxidative stress, *Lactobacillus johnsonii/gasseri* showed greater tolerance to hydrogen peroxide when cultivated under respiratory conditions, specifically in the presence of oxygen, heme, and menaquinone, than under aerobic conditions, highlighting the dependence of stress resilience on metabolic state (Maresca et al. 2018).

Cross‐protection is another strategy that can enhance a microorganism's resistance to multiple types of environmental stress by preconditioning with a single stressor. This approach leverages the interconnectedness of stress response pathways, whereby adaptation to one type of stress can confer tolerance to others. Shin et al. (2019) demonstrated that heat preconditioning markedly enhanced the tolerance of *Enterococcus faecium* HL7 to acid, ethanol, and alkali challenges, indicating a robust cross‐protective effect (Table 3). Notably, for *Lpb. plantarum* 299v, prior exposure to sublethal temperature, acidic pH, or hydrogen peroxide did not affect survival immediately after spray‐drying, but it improved long‐term survival during storage at room temperature in a condition‐dependent manner, underscoring the need to tailor pretreatment protocols to downstream drying and storage parameters (Barbosa et al. 2015). Consistent with Table 3, Chen et al. (2017) documented cross‐protective gains such as a 46.6‐fold higher heat tolerance after bile preconditioning and a 5.9‐ to 9.6‐fold higher cold tolerance after acid or bile exposure. Prasad et al. (2003) further demonstrated that preconditioning improved long‐term desiccation tolerance of *Lcb. rhamnosus* HN001 during storage, with both heat and osmotic pretreatments markedly reducing viability losses (Table 3).

Although Table 3 compiles diverse attempts to exploit preconditioning and cross‐protection strategies, the outcomes remain highly strain‐specific and vary depending on the stressor type, intensity, and subsequent processing conditions. These findings indicate that although preconditioning can induce cross‐protective effects and represents a promising approach to enhance probiotic robustness, its practical benefits are not universal. Instead, they require careful tailoring of pretreatment protocols to the physiological characteristics of each strain and to the demands of downstream applications such as drying or long‐term storage. Therefore, for successful industrial implementation, preconditioning strategies must be selectively adapted and further modified to balance efficacy, feasibility, and scalability.

Overall, optimizing condition‐specific preconditioning protocols can markedly enhance the environmental adaptability of probiotic strains without relying on molecular genetic modifications. Their integration into industrial processes, such as spray‐drying and freeze‐drying, directly contributes to improving product functionality and stability. Therefore, the scientific refinement of strain selection and pretreatment strategies is a key avenue for developing high‐value functional foods.

### 3.3 Interactive Effects of Multiple Stressors

Most probiotic screening studies have traditionally assessed strain viability under single‐stressor conditions such as low pH, bile, heat, or desiccation (Papadimitriou et al. 2016; Sionek et al. 2024). Although this approach provides essential baseline information, it does not adequately reflect the multifactorial challenges encountered during food processing and gastrointestinal transit (Papadimitriou et al. 2016; Sionek et al. 2024). For instance, acid and bile tolerance remain the most fundamental and widely applied criteria in gastrointestinal survival assessments, whereas in industrial contexts, stressors such as heat, osmotic, and oxidative pressures during spray‐drying, fermentation, and storage are often examined individually (Papadimitriou et al. 2016; Sionek et al. 2024). However, in actual manufacturing, these stressors rarely act in isolation. During the transition from fermentation to freeze‐drying, probiotic cells are exposed simultaneously to variations in temperature, moisture content, and desiccation, with moisture loss also inducing osmotic changes that further challenge cell integrity. Similarly, during storage, multiple parameters, such as temperature, humidity, and oxygen concentration, act concurrently, influencing long‐term stability in a manner that cannot be explained by single‐factor models.

The interaction between combined stressors can be either synergistic or antagonistic. Co‐exposure to acid and bile often results in greater reductions in viability than either condition alone, whereas sublethal heat preconditioning may improve resistance to subsequent desiccation through cross‐protection mechanisms (Gao et al. 2022; Yang et al. 2021). Moreover, osmotic and oxidative stresses encountered during storage may cumulatively exacerbate membrane damage, emphasizing the need for multifactorial evaluation frameworks in predicting shelf stability (Sionek et al. 2024). Therefore, identifying industrially robust probiotic strains requires the adoption of multifactorial stress models that better simulate real‐world conditions. Future research should prioritize the integration of such models into screening pipelines. Advanced tools such as omics‐based profiling and computational modeling can help elucidate the complex regulatory networks governing simultaneous stress responses (Papadimitriou et al. 2016; Gao et al. 2022). Incorporating these approaches would enhance the predictive validity of laboratory assays and support the development of probiotic formulations with genuine industrial robustness.

Taken together, probiotic robustness arises from the combined influence of innate resistance, acquired adaptations, and the interactive effects of multiple stressors. Innate resistance reflects the natural tolerance of strains to environmental challenges such as heat, acid, bile, oxidative stress, osmotic shifts, desiccation, and nutrient limitation. Acquired adaptations, induced through preconditioning or repeated exposure, can further strengthen survival and even confer cross‐protection. However, as industrial and physiological environments involve simultaneous stresses, single‐factor assessments are insufficient. Multifactorial screening strategies are therefore essential to identify strains that can maintain stability and functionality under real‐world conditions. As illustrated in Figure 5, this screening stage represents only the beginning of a broader development pipeline that also includes safety verification and economic feasibility assessments. Whole‐genome sequencing and compliance with regulatory frameworks are indispensable for confirming strain safety, whereas careful cost–benefit considerations are necessary to ensure sustainable commercialization. By embedding stress‐resistance screening within this integrated pipeline, the future of NGPs can be secured.

## 4 Advanced Protection Technologies

Ensuring the optimal survival of probiotic strains during industrial processing demands the selection and application of appropriate drying techniques in combination with the use of protective agents. Drying serves as a key step in reducing water activity, extending shelf life, and enhancing formulation flexibility. However, drying simultaneously imposes substantial stress on microbial viability due to exposure to high temperatures, desiccation, and oxygen. Therefore, effective preservation of probiotic functionality requires an integrated approach in which both the drying methodology and the protective matrix are precisely tailored. Protective agents, often derived from carbohydrates, proteins, or lipids, play crucial roles by forming stabilizing matrices that buffer against heat, acid, and oxidative damage (Vijayaram et al. 2024).

One widely employed strategy to enhance strain robustness is microencapsulation, which physically entraps cells within biocompatible materials. As summarized in Table S3, diverse encapsulation techniques have been applied to improve probiotic survival under industrially relevant stresses, including acid and bile exposure, spray‐drying, freeze‐drying, and long‐term storage. Reported improvements vary from modest twofold gains in digestive tolerance to more than 100‐fold increases in storage stability, depending on the matrix and protective agents employed. Examples include trehalose‐based formulations that markedly reduced viability loss during spray‐drying, micellar casein‐sucrose systems that minimized freeze‐drying damage, and goat milk matrices that enhanced the long‐term stability of *B. animalis* BB‐12. These cases highlight that encapsulation efficacy is highly strain‐ and condition‐specific, but they collectively demonstrate the critical role of optimized protective matrices in extending probiotic shelf life and functional delivery.

Instead of describing each drying process in detail, this section emphasizes the relative strengths, weaknesses, industrial feasibility, and cost considerations of commonly applied microencapsulation techniques. As depicted in the second stage of Figure 5, drying and formulation constitute a critical juncture in the probiotic pipeline, where industrial equipment and processing technologies directly influence viability and functionality. By revisiting Figure 5 in this context, it becomes clear that no single drying or encapsulation method provides a universally optimal solution. Instead, process selection must be guided by a balance between protective efficacy, scalability, regulatory acceptance, and economic viability. This reinforces the importance of tailoring equipment choice and encapsulation strategies not only to strain‐specific sensitivity but also to the demands of downstream storage and delivery.

### 4.1 Physical Microencapsulation Techniques and Matrices

#### 4.1.1 Spray‐Drying

Spray‐drying is one of the most widely used microencapsulation techniques incorporated into the industrial production of probiotics due to its cost efficiency, scalability, and well‐established processing technology (Frakolaki et al. 2021; Barbosa et al. 2015; Agriopoulou et al. 2023). Its major drawback is the strong thermal and osmotic stress, which often requires protective agents such as trehalose or proteins to minimize cell death. For instance, the inclusion of trehalose significantly reduced the viability loss in LGG during spray‐drying, reducing viability loss from 5 to just 0.12–0.26 log CFU/g (Broeckx et al. 2017). Recent formulations combining whey protein concentrate, pullulan, trehalose, and glutamate improved *Lpb. plantarum* ATCC 8014 survival and yielded uniform particle morphology (Sun et al. 2020), further illustrating the necessity of matrix optimization. The addition of tuna oil also improved the post‐drying survival of *Lcb. casei* 431 by 17% under spray‐drying conditions and by 10% during freeze‐drying (Eratte et al. 2015). Overall, spray‐drying remains the most economically feasible method for large‐scale probiotic powders, though optimization is essential for heat‐ and oxygen‐sensitive strains.

#### 4.1.2 Freeze‐Drying

Lyophilization, or freeze‐drying, is a widely employed technique for preserving heat‐sensitive biological products, including probiotics. Although the process is relatively costly, it significantly enhances the long‐term stability and shelf life of the final products. Cryoprotectants such as trehalose, sucrose, or proteins are essential to reduce ice damage (Bodzen et al. 2021; Dianawati et al. 2016). Its key advantage lies in preserving functionality for years, but industrial throughput and cost efficiency remain limiting factors, restricting its use largely to high‐value or premium formulations. For instance, *Lpb. plantarum* encapsulated in a rice protein–fructooligosaccharide matrix‐maintained viability during 180 days at 4°C, with functionality largely preserved even at 30°C for 90 days (Savedboworn et al. 2019). Similarly, optimization of freezing conditions and protective agents such as trehalose combined with skim milk markedly improved the survival of LGG, whereas inappropriate media (e.g., PBS) caused dramatic losses (Wang, Wu, et al. 2025). These findings demonstrate that despite higher cost, freeze‐drying is strategically chosen for premium probiotic formulations because it minimizes thermal stress and ensures long‐term stability when coupled with tailored cryoprotectants and storage conditions.

#### 4.1.3 Fluidized Bed Coating

This technique provides uniform coating, low processing temperature, and good stability but requires specialized equipment and higher operational costs (Lim et al. 2021). It is best suited for controlled‐release applications rather than bulk powders. In practice, fluidized‐bed coating has been shown to enhance the flowability and surface protection of spray‐dried probiotic powders. For example, coating LGG increased the mean particle size four‐ to fivefold (96–141 µm) and significantly reduced fines while maintaining viable counts around 10⁸ CFU/g (Lim et al. 2021). These improvements demonstrate its value as a post‐processing step. However, the technique is not commonly used as a primary drying method because it requires preformed cores, adds process complexity, and increases operational cost. Consequently, fluidized‐bed coating is primarily applied in combination with spray‐drying to improve powder handling and incorporate functional multilayer coatings.

#### 4.1.4 Electrostatic Extrusion

Extrusion is simple and low‐cost, forming gel beads in a CaCl_2_ bath (Rojas‐Muñoz et al. 2023). However, large particle size and poor scalability limit industrial application, restricting its use to experimental or small‐batch products. Indeed, extrusion and ionic gelation provide a mild encapsulation environment for heat‐sensitive strains, but the resulting beads are typically 0.5–3 mm in size, with poor uniformity and low throughput. These limitations hinder large‐scale adoption despite high cell survival during processing. Recent efforts, such as vibrational nozzle technology, have optimized bead morphology and viability in *Limosilactobacillus (Lm.) fermentum* K73 (Rojas‐Muñoz et al. 2023), yet these remain lab‐scale optimizations rather than industrial solutions. Thus, extrusion‐based encapsulation retains value for research applications and customized products requiring targeted release but has not achieved widespread commercialization due to scalability and sensory limitations.

### 4.2 Chemical Microencapsulation Techniques

#### 4.2.1 Microencapsulation via Coacervation

Coacervation is effective for enhancing probiotic stability under low pH and during storage.

Complex coacervation reinforced with transglutaminase improved survival of *L. acidophilus* in acidic juices for 63 days, with greater stability in orange juice compared to apple juice (da Silva et al. 2021). Synergistic biopolymers, such as alginate, pectin, chitosan, and carrageenan, enable pH‐responsive release, whereas novel polysaccharides like botryosphaeran improve *Lcb. casei* viability at 5°C under simulated GI stress (Xu et al. 2024).

Plant protein‐based microcapsules provide sustainable, biodegradable alternatives capable of encapsulating both hydrophilic and hydrophobic cargos (Dinh et al. 2024). Despite their advantages, reproducibility, raw material cost, and regulatory acceptance remain major industrial barriers.

#### 4.2.2 Interfacial Polymerization

Interfacial polymerization (IP) creates tailored, multi‐layered membranes at oil–water interfaces, offering thermo‐responsive and pH‐sensitive release.

Recent innovations include PLL‐g‐PNIPAM complexes enabling heat‐triggered release (Sixdenier et al. 2021), hydrogen‐bonded films dissolving at neutral pH (Dupré de Baubigny et al. 2017), and plant protein capsules fabricated by microfluidics with ∼98% biodegradability (Dinh et al. 2024). Layer‐by‐layer IP on single cells also improved gastric survival and intestinal adhesion (Xiong and Sun 2024).

Nevertheless, industrial use is limited by complex processing, solvent use, and regulatory hurdles, confining IP to experimental or pilot‐scale systems.

### 4.3 Physicochemical Microencapsulation Techniques

#### 4.3.1 Emulsification/Ionic Gelation

The combination of emulsification and ionic gelation is a commonly employed and simple technique for the encapsulation of probiotics and is characterized by the absence of extreme temperatures or organic solvents (Varnaitė‐Kapočė et al. 2025).

This technique yields high survival under gastrointestinal simulation but often produces beads of 0.5–3 mm, which limit sensory acceptance and scalability (Sin et al. 2025). Recent efforts using vibrational nozzle technology have improved bead uniformity and viability (Rojas‐Muñoz et al. 2023), but applications remain largely confined to research or niche functional foods.

#### 4.3.2 Emulsion Spraying

Emulsion spraying, commonly associated with spray‐drying, represents a scalable and efficient method for the production of powdered microcapsules (Yin, Chen, et al. 2024). The production of large quantities of microcapsules with satisfactory shelf stability is effective; however, the thermal stress during spray‐drying may adversely affect probiotic viability. Thus, although emulsion spraying is closer to industrial feasibility than ionic gelation, its reliance on high temperature drying necessitates careful wall material selection and process optimization (Farahmand et al. 2024; Wang, Ding, et al. 2025; Yin, Chen, et al. 2024).

#### 4.3.3 Supercritical Fluid Technology (SCF)

SCF using CO_2_ is eco‐friendly and solvent‐free, yielding uniform particles with minimal heat damage (Mamvura et al. 2011; Wang et al. 2021). Common methods include precipitation with compressed anti‐solvent (PCA) and particles from gas‐saturated solutions (PGSS) (Thantsha et al. 2014; Thantsha et al. 2009). SCF methods allow fine control of particle size and morphology without solvent residues, aligning with sustainability goals. However, the high capital investment and limited industrial precedent restrict their application mostly to pharmaceuticals or high‐value nutraceuticals rather than mainstream probiotic foods.

The aim of any physicochemical microencapsulation technique is to create a protective barrier for probiotics that will enhance their survival during processing, storage, and transit through the gastrointestinal environment, thereby improving their efficacy and targeted delivery (Li et al. 2023; Thantsha et al. 2014; Yin, Chen, et al. 2024). As summarized in Table 4, scalability and regulatory feasibility remain critical bottlenecks for probiotic encapsulation technologies. Emulsification and ionic gelation methods, although limited by bead heterogeneity, have been widely adopted due to their mild conditions and low‐to‐medium costs, which make them attractive for functional food applications (Gao, Ma, et al. 2022; Li et al. 2023). Spray‐drying, despite concerns about thermal inactivation, continues to dominate the probiotic industry because of its high throughput, cost‐efficiency, and compatibility with established manufacturing infrastructure (Farahmand et al. 2024; Wang, Ding, et al. 2025). By contrast, supercritical fluid approaches remain largely restricted to laboratory and pilot‐scale applications, primarily because of high equipment costs, limited literature supporting probiotic‐specific outcomes, and uncertainties surrounding regulatory acceptance (Mamvura et al. 2011; Thantsha et al. 2014). These considerations underscore that comparative evaluation of encapsulation methods must extend beyond protective efficacy to address industrial feasibility, economic viability, and compliance with food and pharmaceutical regulatory frameworks.

**TABLE 4:** Comparison of physicochemical microencapsulation techniques for probiotics.

Feature	Emulsification/Ionic gelation	Emulsion spraying (spray‐drying)	Supercritical fluid technology
**Core principle**	Formation of emulsion, followed by ionic cross‐linking to generate gel matrix (Gao et al. 2022; Li et al. 2023; Pinto et al. 2025)	Atomization of emulsion in heated air for expedited solvent evaporation (Farahmand et al. 2024; Wang, Ding, et al. 2025; Yin, Chen, et al. 2024)	Use of supercritical CO_2_ as a solvent/anti‐solvent for particle precipitation (Mamvura et al. 2011; Thantsha et al. 2014; Thantsha et al. 2009)
**Typical materials**	Polysaccharides (alginate, xanthan gum, gellan gum, chitosan, carrageenan) and proteins	Polysaccharides (maltodextrin, gum Arabic, starch, PVA, alginate) and proteins (whey)	Polymers (e.g., poly lactic acid, poly‐(vinylpyrrolidone)‐poly‐(vinylacetate‐*co*‐crotonic acid)
**Key advantages**	Mild conditions, cost‐effective, high cell retention, small particle diameter, industrially scalable	High efficiency, fast, simple, cost‐effective, stable powders, reduced cold chain needs, multi‐functional	Low environmental impact, no organic solvents, high purity, precise particle control
**Key limitations**	Emulsion instability, inhomogeneity, organic solvent removal challenges, alginate susceptibility	High temperatures can impact viability, challenges in nanoencapsulation, material availability issues	Primarily research phase, limited literature for probiotics, high equipment costs, potential damage to biologicals
**Scalability**	High	High	Medium
**Impact on probiotic viability**	High (mild conditions)	Moderate (requires optimization for temperature)	High (mild conditions, but process effects need investigation)
**Relative cost**	Low to medium	Low to medium	High (due to equipment)

Taken together, advanced protection technologies for probiotics demonstrate that no single method can simultaneously optimize cost‐efficiency, scalability, and functional performance. Physical methods such as spray‐ and freeze‐drying remain the industrial backbone due to feasibility and established infrastructure, whereas chemical and physicochemical approaches provide enhanced protection but face challenges related to reproducibility, regulatory acceptance, and economic viability. Hybrid strategies and multi‐layered encapsulation systems are increasingly emerging as practical solutions, aiming to combine the strengths of different methods while minimizing their respective drawbacks. This reinforces the conclusion that the choice of encapsulation or drying strategy must be context‐specific, guided not only by strain sensitivity and intended application but also by industrial scalability, consumer acceptance, and evolving regulatory frameworks, rather than by the search for a universal “best” technique.

## 5 Regulatory Frameworks for Probiotic‐Based Functional Foods Across Countries

Ensuring the successful development and commercialization of probiotic‐based functional foods in the global marketplace requires the systematic preparation of scientific dossiers that meet the functional claim requirements established by each country's regulatory authorities. A foundational milestone in this context was the publication of the *Guidelines for the Evaluation of Probiotics* by the FAO and the WHO in 2002. This FAO/WHO document provided internationally recognized safety and efficacy standards, and individual countries subsequently formulated their own national policies and regulatory frameworks for probiotic products. Moreover, academic organizations such as the International Scientific Association for Probiotics and Prebiotics (ISAPP) have continued to contribute as scientific navigators by offering evidence‐based guidance. Although ISAPP does not participate directly in legislative or regulatory processes, its recommendations, such as the 2024 consensus statement on the co‐administration of probiotics and antibiotics, support scientific dialog and contribute to improved regulatory coherence across regions. Nevertheless, the definitions of functional foods, classification systems, claim approval processes, evaluation methodologies, and levels of scientific substantiation required still vary significantly across jurisdictions. Therefore, a single universal regulatory strategy is often inadequate. Instead, the adoption of a tailored approach is necessary to reflect the specific regulatory expectations of each country.

**FIGURE 6:**
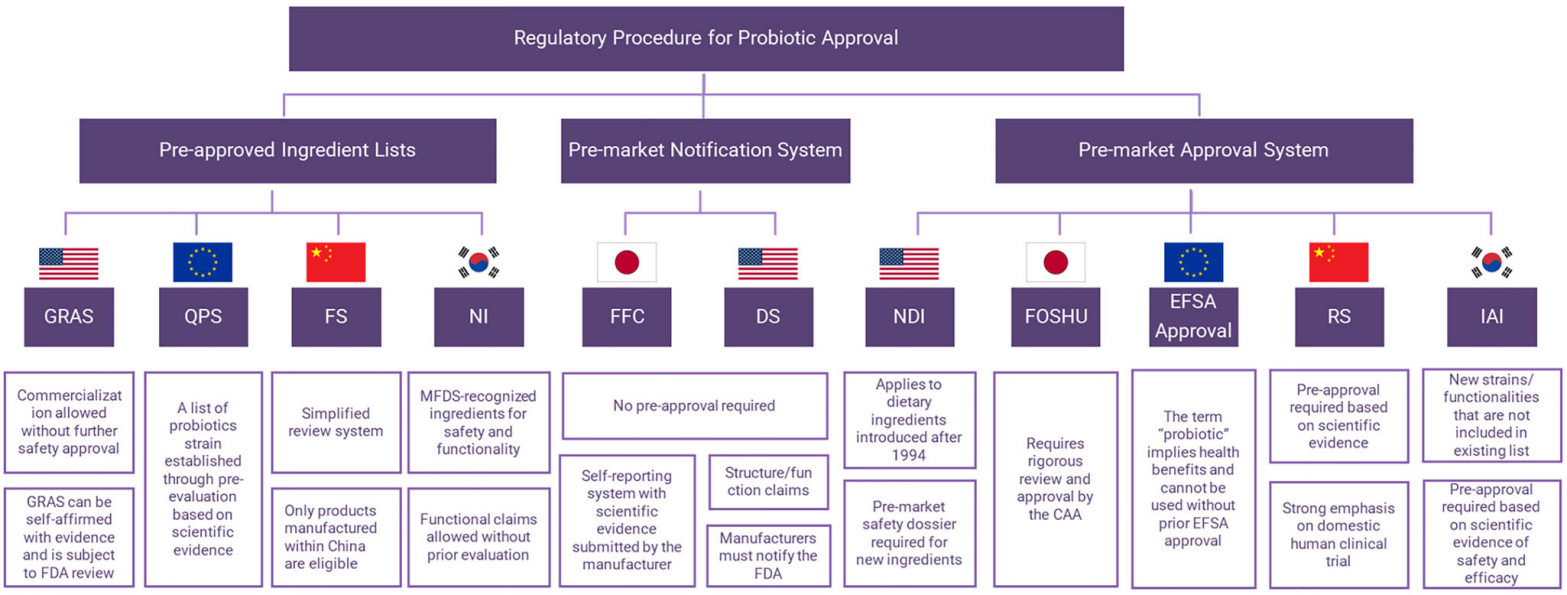
Comparative overview of national regulatory pathways for probiotics functional foods. DS, dietary supplements; FFC, foods with function claims; FOSHU, foods for specified health uses; FS, filing system; GRAS, generally recognized as safe; IAI, individually approved ingredients; NDI, new dietary ingredient; NI, notified ingredients; QPS, qualified presumption of safety; RS, registration system.

The aim of this section is to compare and analyze the regulatory heterogeneity and institutional particularities of probiotics use across major markets. Table S4 provides a structured overview of key regulatory components by country, including category designations, review mechanisms, and the permissible scope of functional claims. To further support strategic planning for global market entry, Figure 6 visualizes the regulatory pathways and critical distinctions among national systems, offering an intuitive summary of the essential regulatory considerations that must be addressed when targeting international markets.

### 5.1 Republic of Korea (Ministry of Food and Drug Safety, MFDS)

In Korea, probiotics are classified as functional ingredients under the Health Functional Food Act, and their regulatory approval follows two main pathways: notified (pre‐approved) ingredients (NI) and individually approved ingredients (IAI). Notified ingredients refer to substances whose safety and functionality have already been recognized by the MFDS. Products containing these ingredients, when formulated in accordance with the specified standards and intake levels, may make functional claims without additional evaluation. In contrast, individually approved ingredients involve new strains or functionalities that are not included in the existing list. For such cases, manufacturers are required to submit scientific evidence demonstrating the safety and efficacy of the ingredient, and this evidence then undergoes a comprehensive individual review by the MFDS.

The required documentation for individual approval includes taxonomic identification of the strain. Genetic characterization (e.g., 16S rRNA sequencing, MLST, or whole‐genome sequencing) is recommended or required on a case‐by‐case basis. In addition, safety assessments must address features such as toxicity and antibiotic resistance. Evidence of functionality should be supported by mechanistic studies using both in vitro and in vivo models, as well as statistically significant findings from human intervention trials. Functional claims must be substantiated by clinical data, whereas in vitro and animal studies provide supportive evidence for the proposed mechanism. To ensure safety, probiotic strains must pass specific assessments, including tests for antibiotic resistance, hemolytic activity, cytotoxicity (e.g., LDH release), and metabolic characteristics (e.g., d‐lactate production and BSH activity) (MFDS 2022). The MFDS provides dedicated guidelines for both safety and functionality evaluation of probiotics, and the scientific rigor and consistency of submitted evidence are subject to strict review.

### 5.2 China (National Medical Products Administration, NMPA)

In China, probiotic products are regulated as “health foods” under the so‐called blue hat system, and their approval is generally granted at the strain‐specific level, in a framework comparable to Korea's NI. China employs a dual‐track regulatory framework consisting of the registration system (RS) and the filing (notification) system (FS), which is determined by factors such as whether the probiotic strain is included in the pre‐approved raw materials directories, whether the product is imported, and whether the claimed health function is among those already listed. Any unregistered strain must follow a mandatory new registration process prior to any use in health food formulations (CIRS 2023). Functional claims must be selected from a government‐designated list of 24 approved health functions, which include modulation of the intestinal microbiota, promotion of digestion, and alleviation of constipation, among others (CIRS 2023). The documentation required for new strain registration includes accurate taxonomic identification and characterization of the strain, results of safety assessments, data from human clinical trials conducted either within China or abroad, and analytical test reports issued by certified laboratories. Safety evaluations must address antibiotic resistance, hemolytic activity, potential toxin production, and the presence of toxin‐related genes. Additional biological characterization includes assessments of acid and bile tolerance, adhesion to intestinal epithelial cells, and anti‐pathogenic activity. As in Korea, functional substantiation requires comprehensive evidence, although the depth of in vitro, animal, and human studies varies depending on the type of claim. Chinese authorities place stronger emphasis on clinical trial data generated domestically, and foreign data alone may sometimes be considered insufficient to support functional claims.

In addition to the RS, China also operates the FS for probiotics entering the market as health foods. Introduced in 2016, this system is applicable only to domestically manufactured products using probiotic strains and functional claims included in the pre‐approved directories published by the NMPA. Compared to the RS, the FS offers a simplified review process, reduced timelines, and lower data submission burdens, as it does not require clinical or toxicological testing. However, the probiotic strains and permissible functional claims are strictly limited to those specified in the NMPA's official list. New strains are not listed, and products manufactured overseas remain subject to the full registration process. This dual‐track regulatory framework is regarded as a harmonized approach that balances rigorous scientific validation of probiotic safety and efficacy with the need to facilitate timely market access for industry stakeholders in China.

### 5.3 United States (FDA)

In the United States, probiotic products are generally classified as dietary supplements and are regulated under the Dietary Supplement Health and Education Act (DSHEA) enacted in 1994 (U.S. Food and Drug Administration 2024a). However, depending on intended use and claims, certain probiotics may also be regulated as conventional foods under GRAS or as live biotherapeutic products under the drug framework. Under this framework, dietary supplements are prohibited from making claims related to the prevention, treatment, or mitigation of diseases. Instead, they are limited to structure/function claims, which describe the supplement's effect on the normal structure or function of the human body. Examples of structure/function claims are statements such as “supports digestive health” or “improves the intestinal environment.” These claims do not require pre‐approval from the FDA; however, manufacturers are obligated to notify the FDA within 30 days of marketing such claims. Additionally, product labels must include the following disclaimer:

This statement has not been evaluated by the Food and Drug Administration. This product is not intended to diagnose, treat, cure, or prevent any disease.

Manufacturers are responsible for substantiating any structure/function claims with competent and reliable scientific evidence, which must be readily available in case of regulatory audits or legal disputes. Accordingly, companies intending to launch probiotic products in the US market must avoid disease‐related claims and focus on statements consistent with bodily structure or function and supported by appropriate scientific data.

In addition to DSHEA, the US regulatory framework includes the GRAS and NDI notification systems. The GRAS designation pertains to substances that are widely accepted as safe based on scientific data and expert consensus (U.S. Food and Drug Administration 2023). Although manufacturers may self‐declare GRAS status based on available evidence, submitting a GRAS Notice to the FDA enables a formal review and the issuance of a “no questions” letter. GRAS recognition primarily applies to conventional foods, and its applicability to supplements may depend on intended use and labeling. Conversely, the NDI notification system applies to dietary ingredients that were not marketed in the United States prior to October 15, 1994. In such cases, manufacturers must submit a pre‐market safety dossier for the new ingredient, which the FDA reviews within 75 days (U.S. Food and Drug Administration 2024b). For probiotics, this distinction is particularly important, as strain‐specific physiological properties and safety profiles often necessitate individualized evaluations. When a probiotic strain is not covered under GRAS, the NDI pathway often becomes a critical route for establishing safety and facilitating legal market entry.

Both GRAS and NDI frameworks are essential tools for assessing the regulatory acceptability of probiotic ingredients. For novel strains or those without a recognized history of safe use in the United States, fulfilling NDI requirements may represent a pivotal determinant in successful commercialization, whereas strains developed as live biotherapeutics must instead follow the drug approval pathway.

### 5.4 European Union (EFSA)

In the European Union (EU), probiotics are generally regulated as food supplements. However, when a manufacturer intends to display a health claim on its labeling, the product must undergo prior evaluation by the EFSA and receive final authorization from the European Commission, in accordance with the Nutrition and Health Claims Regulation (NHCR). This requirement is based on Regulation (EC) No. 1924/2006, which governs health claims made on food products (European Parliament and Council 2006). The EFSA conducts rigorous assessments of probiotic‐related health claims, focusing on the quality, consistency, and scientific validity of the supporting evidence. Importantly, the use of the term “probiotic” itself is not officially authorized in the EU as a generic descriptor, as EFSA has interpreted it as an implicit health claim. As such, it cannot appear on product labels without prior EFSA approval (Salminen et al. 2021). To obtain authorization for a probiotic health claim, the following conditions must be met: (1) accurate taxonomic identification and characterization of the strain, (2) evidence from well‐designed human intervention studies meeting EFSA's stringent criteria (EFSA Panel on Dietetic Products, Nutrition and Allergies (NDA) 2011), (3) evidence demonstrating the strain's viability during product shelf life and its stability during digestion, and (4) a scientifically substantiated explanation of the proposed mechanism of action.

To date, most health claims submitted to EFSA concerning probiotics have been rejected, primarily due to insufficient substantiation. In response, recent research has shifted toward the precise elucidation of strain‐specific functionalities and mechanisms, supported by randomized, double‐blind, placebo‐controlled human studies to generate robust evidence.

In parallel, EFSA applies the qualified presumption of safety (QPS) approach to evaluate the safety of microbial strains used in food and feed. QPS offers a pre‐evaluation framework based on scientific evidence at the species level, although strain‐specific qualifications may be required in certain cases. The granting of QPS status to a microorganism requires documentation detailing its taxonomic identification, absence of pathogenicity and toxigenicity, antibiotic resistance profiles, and historical usage data. Although most core probiotic species, such as *Lpb*. (formerly *Lactobacillus*) *plantarum*, *Bifidobacterium* spp., and certain *Bacillus* species, are listed in the QPS catalog, newly isolated or genetically modified strains require independent safety evaluations. Therefore, manufacturers seeking the entry of a probiotic into the EU market must provide comprehensive, strain‐specific safety data as a prerequisite, regardless of its QPS status. Establishing this scientific evidence is critical for regulatory compliance and market acceptance in the European context.

### 5.5 Japan (Consumer Affairs Agency, CAA)

In Japan, health supplements containing probiotics are categorized into three main groups: Foods for Specified Health Uses (FOSHU), Foods with Nutrient Function Claims (FNFC), and Foods with Function Claims (FFC). As FNFC applies only to vitamins and minerals with pre‐approved nutrient‐function statements, it is not relevant to probiotic products and will not be discussed further here. Both FOSHU and FFC are the key systems directly related to probiotic products. Both systems require scientific evidence that substantiates the functionality and safety of the products, but they differ in terms of their application procedures and regulatory intensities.

FOSHU is a system that requires rigorous review and formal approval by the Japanese government (CAA Japan 2023a). To obtain FOSHU certification, manufacturers must provide scientific evidence that their products effectively deliver specific health benefits. The submission materials include human intervention study results, long‐term safety data, and explanations of the mechanism of action. For probiotic products, strain‐specific safety and functionality assessments are required, including pathogenicity, toxicity, antibiotic resistance, and potential adverse effects from metabolic byproducts. Japan has no official strain safety list equivalent to the US GRAS or EU QPS; therefore, the applicant must provide safety substantiation for each strain used. If the aim is to claim health benefits, such as improving gut health or modulating immunity, the product must undergo these stringent safety and functionality assessments to obtain FOSHU approval and display the functional claim.

The FFC system, introduced in 2015, is a pre‐market notification scheme managed by the CAA (CAA Japan 2023b). In this system, manufacturers can submit their scientific evidence of functionality, such as summaries of relevant studies, safety data, and label designs, to the CAA at least 60 days prior to marketing. Unlike FOSHU, FFC does not involve official government “approval”; instead, the responsibility for scientific substantiation and product safety lies entirely with the manufacturer, whereas the CAA publishes the notification and assigns a reference number. Makers of probiotic products can utilize the FFC system to make certain claims, such as improving gut health or enhancing bowel movements, but the supporting evidence is expected to be specific to the strain or product used. Regarding safety, although FFC does not have explicit regulations at the strain level, a scientific safety summary that includes the characteristics, safety, and previous consumption history of the microbial strains used in the product must be submitted. Therefore, securing strain‐specific safety data is essentially a prerequisite, particularly for new strains, and verifying their safety with proprietary data is crucial.

### 5.6 Critical Cross‐Regional Analysis and Economic Considerations

A comprehensive evaluation shows that the substantiation of the functionality and safety of probiotic products depends fundamentally on a detailed analysis of country‐specific regulatory requirements and the preparation of scientifically robust evidence demonstrating strain‐specific functionality and safety. Successful international market entry demands a tailored strategy that includes the design of human intervention trials and the collection of supporting literature in alignment with each jurisdiction's regulatory framework. To further support strategic planning for such global market entry, Figure 6 visualizes the regulatory pathways and critical distinctions among national systems, providing an intuitive overview of the essential considerations that must be addressed.

Nevertheless, identical experimental outcomes may still lead to divergent regulatory decisions across countries, increasing overall complexity, time, and cost. For example, probiotic foods and supplements are categorized differently in various regions, and this categorization is directly linked to variable requirements and costs across development stages (Spacova et al. 2023).

Moreover, regulatory evaluation methodologies contain notable gaps. Current challenges in standardization, such as the validation of microbial identity, potency, and stability, contribute to extended approval timelines and elevated development burdens (Cordaillat‐Simmons et al. 2020).

Importantly, such regulatory heterogeneity influences strain selection and commercialization strategy. Companies may favor widely established strains like *Lpb. plantarum* 299v to minimize regulatory risk, but this tendency narrows consumer options and hinders market access for novel next‐generation strains.

For NGPs, regulatory barriers intensify. These products often face unclear legal classification, stringent safety requirements such as whole‐genome sequencing and virulence or antibiotic resistance assessment, and substantial clinical validation burdens in different jurisdictions (Vijayaram et al. 2024). In addition, manufacturing and technological limitations, such as poor oxygen tolerance in many NGP strains, pose practical obstacles to commercialization and increase production costs (Lalowski and Zielińska 2024).

Therefore, future regulatory discourse should integrate not only scientific and safety benchmarks but also systematic evaluation of economic and temporal burdens. Comparative research that delineates both shared and divergent regulatory expectations across markets is essential for guiding policymakers and industry stakeholders toward strategies that protect public health without impeding innovation.

## 6 Future Perspectives

### 6.1 Artificial Intelligence (AI)‐Driven Strain Screening and Predictive Modeling

Artificial intelligence and machine learning (ML) are reshaping probiotic strain discovery by enabling rapid and systematic early stage screening. MetaProbiotics is a recent example, applying language models and random forest algorithms to metagenomic data and successfully identifying probiotic‐related bins from clinical samples, including those do not present in the training dataset (Wu et al. 2024). Such results demonstrate how AI can accelerate the transition from large‐scale metagenomic sequencing to candidate prioritization. Future progress, however, will require broader validation using standardized clinical and industrial datasets to ensure reproducibility and external applicability.

The Integrated Lactic Acid Bacteria Database (iLABdb) aggregates genomic data specific to LAB, providing a practical resource for simultaneously screening functional genes and safety markers (Jin et al. 2023). While highly useful for LAB‐focused studies, its limited scope underscores the need for broader coverage of NGPs such as *Bifidobacterium* and *Akkermansia*. Expanding the taxonomic range and integrating phenotype‐linked metadata would make such resources more impactful for industrial strain development.

Newer platforms, including iProbiotics, Probio‐Ichnos, and ProbioMinServer, add further layers of predictive capability, from whole‐genome‐based trait analysis to curated literature and integrated safety assessment (Sun et al. 2022; Liu et al. 2023; Tsifintaris et al. 2024). These resources highlight an emerging ecosystem of computational tools that can shorten the gap between genome sequencing and practical evaluation. Yet their adoption will depend on validation in industrially relevant contexts and interoperability with existing pipelines.

Conventional resources remain essential as well. The Protein Families Database (Pfam), the Clusters of Orthologous Groups (COG) database, and the Comprehensive Antibiotic Resistance Database (CARD) continue to provide foundational frameworks for gene annotation and safety assessment (El‐Gebali et al. 2019; Galperin et al. 2021; Alcock et al. 2023). By contrast, the Antibiotic Resistance Genes Database (ARDB) is no longer updated and now serves primarily as a historical reference (Liu and Pop 2009). In practice, CARD, ResFinder, and MEGARes are the most relevant resources for antimicrobial resistance gene annotation in modern probiotic genomics (Zankari et al. 2012; Doster et al. 2020).

Looking forward, the future of probiotic screening lies in integrated pipelines that merge AI‐driven innovation with actively curated databases. AI can reduce the cost and time required for candidate discovery, but its outputs must be anchored in reliable genomic annotation frameworks to avoid false positives. Dynamic resources that combine predictive modeling with up‐to‐date safety and functionality references will provide the foundation for NGPs that are both effective and compliant with regulatory standards.

### 6.2 High‐Throughput Cultivation and Isolation Platforms

Translating in silico predictions into tangible probiotic candidates requires advanced cultivation systems capable of processing large numbers of isolates simultaneously. High‐throughput isolation platforms, such as the Prospector system, address this need. The Prospector platform developed by Isolation Bio enables the parallel cultivation of microorganisms in thousands of nanoscale microchambers. Through automated imaging, target colonies can be identified and subsequently transferred into 96‐well plates for downstream applications, thereby streamlining isolation and screening workflows (Lynch et al. 2023).

This approach not only enhances the recovery of rare or slow‐growing taxa but also shortens the timeline from environmental sampling to functional characterization. When integrated with AI‐driven genomic screening, these platforms provide a closed‐loop discovery pipeline in which computational predictions guide selective cultivation and the resulting isolates are rapidly tested for viability and function.

This represents a transition from traditional, labor‐intensive isolation methods to scalable strategies that can support both academic research and industrial product development. However, these platforms should be regarded not as tools for direct industrial‐scale production but as enablers of early stage discovery. Their primary value lies in rapidly securing diverse and potentially valuable isolates, which must then undergo downstream process development such as fermentation scale‐up, formulation, and long‐term stability testing to determine commercial applicability. In this way, systems like Prospector accelerate the front end of the research‐to‐industry pipeline rather than serving as endpoints of industrial deployment.

### 6.3 Personalized Probiotics and Precision Nutrition

Personalization has emerged as a key consideration in future probiotic development. Early work demonstrated that postprandial glycemic responses vary widely among individuals and can be predicted using microbiome features (Zeevi et al. 2015). Building on this foundation, randomized trials have shown that dietary fiber can improve glucose metabolism in some individuals through microbiome‐mediated mechanisms such as increased abundance of *Prevotella* (Kovatcheva‐Datchary et al. 2015). More recently, a randomized controlled trial in Nature Medicine reported that a personalized dietary program combining microbiome profiling, postprandial responses, and health history produced greater improvements in cardiometabolic markers compared to standard dietary guidance (Bermingham et al. 2024). Reviews have further emphasized that integrating microbiome, metabolomic, and host phenotype data is central to the future of personalized nutrition and microbiota‐based interventions (Sarfraz et al. 2022).

Looking forward, personalization is likely to converge with NGPs such as *A. muciniphila* and *Faecalibacterium prausnitzii*, which show promise in metabolic and inflammatory disorders. However, bringing such tailored products to market will require well‐powered, multicenter clinical trials and transparent computational workflows. Without such infrastructure, personalized probiotics will remain more of a conceptual opportunity than a practical therapeutic strategy.

### 6.4 Industrial‐Scale Validation of Stability and Encapsulation Strategies

To date, there are very limited published academic reports that systematically evaluate the industrial‐scale validation of probiotic encapsulation technologies, although such validation is likely performed within companies but rarely disclosed. Although it is highly likely that such validation is performed internally by companies, the results are rarely disclosed, creating a significant knowledge gap between laboratory innovation and industrial practice. This lack of shared evidence constrains the broader scientific community's ability to assess which strategies are truly effective under commercial conditions.

Laboratory studies have consistently demonstrated the potential of biomaterials such as alginate, chitosan, and protein‐polysaccharide matrices to improve probiotic survival during processing, storage, and simulated gastrointestinal transit (Agriopoulou et al. 2023; de Deus et al. 2024). For instance, alginate–xanthan gum hydrogels have been shown to enhance the survival of *L. rhamnosus* under simulated gastric stress (Oberoi et al. 2021). These results provide valuable proof‐of‐concept, but long‐term efficacy within commercial distribution chains remains unknown because such data have not been shared in academic publications.

A key limitation of the current literature is the lack of systematic evaluation of economic feasibility and scalability. Academic reports rarely provide data on cost per unit, process throughput, or compatibility with existing production pipelines, and such information is often retained within industry rather than disclosed in peer‐reviewed publications.

Future progress will depend on stronger collaboration between academia and industry. Generating comparative datasets on survival, functional retention, and cost‐effectiveness under real production and distribution conditions will be critical. Only then can encapsulation strategies move beyond laboratory‐scale demonstrations and contribute meaningfully to the industrial development of probiotics and NGPs.

### 6.5 Regulatory Harmonization and Adaptable Frameworks to a Global Standard

Probiotic regulation remains fragmented across regions. In the United States, products are assessed under the GRAS or NDI frameworks, whereas Europe applies the QPS approach. Asia employs a range of systems, including FOSHU and FFC in Japan, RS and FS in China, and NI and IAI in Korea. This heterogeneity complicates global commercialization, because companies must prepare separate dossiers for each jurisdiction.

Complete harmonization is unlikely in the near term, yet the absence of common criteria continues to act as a barrier to international market access. A pragmatic strategy would be to establish modular dossiers that include core safety and efficacy data applicable across regions, with additional modules adapted to local requirements. In practice, companies are often required to submit the same data, such as antimicrobial resistance profiles, separately to both the European QPS system and the U.S. GRAS process. Differences in application formats and procedural requirements create additional administrative burdens and can prolong approval timelines. A forward‐looking solution would be to create an online platform through which validated safety and efficacy datasets can be securely shared among regulatory authorities. Such a system would reduce redundant submissions, save time, and increase transparency, thereby accelerating international commercialization.

The challenge is even more pronounced for NGPs. For these strains, genomic stability, exclusion of toxin genes, and host interaction data remain insufficiently standardized (Abouelela and Helmy 2024). Without consensus on minimal datasets, regulatory uncertainty will continue to limit investment and delay translation from laboratory research to market‐ready products. Global dialogue among regulators, scientists, and industry stakeholders will be essential to design adaptable frameworks that balance scientific rigor with the need for efficiency and market access.

## 7 Conclusion

This review has provided a comprehensive examination of the developmental cycle of probiotics, emphasizing the necessity of a multilayered strategy that integrates functionality, safety, stability, and regulatory considerations. It has outlined how dual‐screening systems, inducible stress resistance mechanisms, and encapsulation‐based stabilization technologies can provide practical foundations for improving the commercial viability of probiotic strains. These approaches are scientifically robust and relevant to industrial applications, offering guidance for enhancing product reliability.

Collaborations between academia and industry were highlighted as essential for connecting strain development, functional validation, and market application. Such cooperative frameworks enable the early identification of high‐performance strains and contribute to the development of products that are both scientifically validated and trusted by consumers. Strategic considerations must extend beyond storage viability to encompass colonization efficiency and functional expression in the host, as these are critical to ensuring both efficacy and consumer confidence.

This review also underscored the importance of regulatory frameworks for facilitating global commercialization. By comparing systems such as GRAS and NDI in the United States, QPS in Europe, and diverse regulations in Asia, it became clear that understanding and navigating these frameworks is essential for advancing probiotics from laboratory findings to market‐ready products.

In summary, probiotics research and development require an integrated approach that addresses multiple dimensions simultaneously. The combined focus on scientific rigor, industrial feasibility, and regulatory alignment provides a solid foundation for the sustainable growth and competitiveness of probiotic‐based health products in an evolving global market.

## Nomenclature

AI: Artificial Intelligence
ARDB: Antibiotic Resistance Genes Database
ALE: adaptive laboratory evolution
CAA: Consumer Affairs Agency
CARD: Comprehensive Antibiotic Resistance Database
COG: Clusters of Orthologous Groups
CFU: colony forming units
DS: dietary supplements
DSHEA: Dietary Supplement Health and Education Act
EFSA: European Food Safety Authority
EU: European Union
FAO: Food and Agriculture Organization of the United Nations
FDA: Food and Drug Administration
FFC: Foods with Function Claims
FNFC: Foods with Nutrient Function Claims
FOSHU: Foods for Specified Health Uses
FS: filing system
GRAS: generally recognized as safe
IAI: individually approved ingredients
IBD: inflammatory bowel disease
IBS: irritable bowel syndrome
IP: interfacial polymerization
ISAPP: International Scientific Association for Probiotics and Prebiotics
LAB: Lactic Acid Bacteria
*Lcb*.: *Lacticaseibacillus*
*Lpb*.: *Lactiplantibacillus*
*L*.: *Lactobacillus*
*Lm*.: *Limosilactobacillus*
MFDS: Ministry of Food and Drug Safety
MIC: minimum inhibitory concentration
ML: Machine Learning
NDI: new dietary ingredient
NGPs: next‐generation probiotics
NI: notified ingredients
NMPA: National Medical Products Administration
PCA: precipitation with compressed anti‐solvent
Pfam: Protein Families Database
PGSS: particles from gas‐saturated solutions
QPS: qualified presumption of safety
ROS: reactive oxygen species
RS: registration system
scCO_2_
: supercritical carbon dioxide
SCF: supercritical fluid
SNP: single nucleotide polymorphism
WHO: World Health Organization

## Author Contributions

**Hye Kim**: conceptualization, methodology, data curation, formal analysis, validation, investigation, visualization, writing – review and editing, writing – original draft. **Ariful Haque**: methodology, investigation, data curation, visualization, writing – review and editing. **Abdur Razzak**: methodology, data curation, visualization, writing – review and editing. **Min Ji Jang**: methodology, data curation, visualization, investigation, writing – review and editing. **Sehyeon Song**: methodology, data curation, investigation, writing – review and editing. **Seockmo Ku**: conceptualization, funding acquisition, writing – original draft, writing – review and editing, project administration, resources, supervision, validation, investigation.

## Conflicts of Interest

The authors declare no conflicts of interest.

## Supporting information




**Supplementary Tables**: crf370320‐sup‐0001‐TableS1‐S4.docx

## Data Availability

Data will be made available on request.
