# Supplementary material for: Probiotic Development Strategy Centered on Stability and Regulatory Considerations
Source: Compr Rev Food Sci Food Saf. 2026 Jan 5;25(1):e70320. doi: 10.1111/1541-4337.70320 (PMC12770815; doi:10.1111/1541-4337.70320)
Supplement: Supplementary file 1 — Supplementary Tables: crf370320‐sup‐0001‐TableS1‐S4.docx [file CRF3-25-e70320-s001.docx]

**Supplementary Table S1 Comparative Overview of Existing Review Articles on Probiotics: Commonly Addressed Themes and Remaining Gaps.**

| Category | Commonly Addressed | Gaps | Key References |
| --- | --- | --- | --- |
| Functionality | GI, metabolic, immunological, neurological health benefits widely reviewed | Shortage of long-term, large-scale clinical trials; insufficient standardized functional indices | T.F.da Silva et al. 2024; Ge et al. 2024; Shah et al. 2024; Petrariu et al. 2024 |
| Safety | International guidelines (FAO/WHO, FDA GRAS/NDI, EFSA QPS); genomic ID; AMR/toxin screening | Limited safety data for next-generation probiotics (NGPs); lack of harmonized global criteria; inadequate evaluation in vulnerable groups | Roe et al. 2022; Spacova et al. 2023; Liang et al. 2024 |
| Stability | Encapsulation, protective agents, stress adaptation strategies | Few practical improvement strategies validated under industrial settings; limited critical analyses of existing commercialization and preservation cases | Q. Lin, Si, et al. 2024; Ge et al. 2024; Wang et al. 2024 |
| Industrial Application | Omics-guided optimization, bioreactor culture, food incorporation, drying technologies | Insufficient critical analyses of successful commercialization cases; lack of economic feasibility evaluation | Yang et al. 2024; Pereira et al. 2024; Liang et al. 2024 |
| Regulation | Regional diversity, international guidelines discussed | Inadequate comparative analyses of regulatory commonalities and differences; lack of concise summary of mandatory evaluation requirements; heavy economic and temporal burdens from heterogeneous frameworks; unclear roadmap for NGP/engineered strains | Roe et al. 2022; Spacova et al. 2023; Aziz 2025; T.F.da Silva et al. 2024 |

Article selection criteria: (i) thematic relevance to our manuscript, (ii) recent publication within the past few years, and (iii) appearance in well-recognized peer-reviewed journals with broad scientific visibility.

**Supplementary Table S2. Comparative Overview of Probiotic Functional Discovery and Commercialization (Full version).**

| Strain (Trade mark) | Type and Origin | In vitro Evidence | In vivo Evidence | Clinical Evidence | Commercialization Status | Product Category | Patent | Reference |
| --- | --- | --- | --- | --- | --- | --- | --- | --- |
| *Lcb. rhamnosus* GG (*LGG ®)* | Conventional, Healthy human feces | Acid/bile tolerance; adhesion to enterocytes; antimicrobial activity | Preclinical evidence supports intestinal mucosa protection and pathogen inhibition | Multiple RCTs: reduction of acute diarrhea, alleviation of IBS/IBD symptoms, immune modulation | Globally established; DSHEA; QPS; | Food (restricted in US); Supplement | Expired patent on strain; Patent on indication and composition | (Capurso 2019; Steele 2022; (NIH) 2021; EFSA BIOHAZ Panel 2025) |
| *B. animalis* subsp. lactis BB-12 (BB-12®) | Conventional, Dairy cultures | Acid/bile tolerance; BSH activity; mucus adhesion | Improved gut barrier, reduced diarrhea in animal models | Multiple RCTs: improved bowel function, prevention of diarrhea, reduced respiratory infections | Globally established; GRAS; QPS | Food; Supplements | Expired patent on strain; Patent on indication and composition | (Isolauri et al. 2011; Jae Ho, Seok Mo, et Seung Hyun 2016; Jungersen et al. 2014; Salminen 2019; Wong, Odamaki, et Xiao 2019; FDA) 2019; EFSA BIOHAZ Panel 2025; Nordström et al. 2021; FDA) 2017; EFSA BIOHAZ Panel 2025) |
| *Lpb. plantarum* 299v (LP299V®) | Conventional, Healthy human intestinal mucosa | GI survival; epithelial adhesion; antimicrobial safety profile | Gut colonization, modulation of local immune markers | RCTs: IBS symptom alleviation, enhanced iron absorption | Widely commercialized; GRAS; QPS | Food; Supplements | Expired patent on strain; Patent on indication | (FDA) 2025; Kaźmierczak-Siedlecka et al. 2020; EFSA BIOHAZ Panel 2025) |
| *Saccharomyces boulardii* CNCM I-745 *(Florastor®)* | Conventional, Peels of tropical fruit | Broad pH and bile tolerance; antimicrobial activity | Protection against H. pylori, prevention of antibiotic-associated diarrhea in animal models | Multiple RCTs: effective in prevention of diarrhea, adjunct therapy for IBD/IBS | Globally established; NDI; QPS | Supplement | Expired patent on strain; Patent on indication and composition | (FDA) 2025; Belzer et al. 2022; Cani et Brochot 2024; Depommier et al. 2019; Yan, Sheng, et Li 2021; Zhang et al. 2025; European 2025) |
| *Akkermansia muciniphila* | NGP, Human gut | Mucin degradation/utilization; outer membrane protein activity | Improved insulin sensitivity, lipid lowering, reduced inflammation in obesity/NAFLD/T2D models | Clinical pilot trials: improved insulin sensitivity and cholesterol profile | Pilot commercialization; NDI (live cell); Novel Food (Pasteurized) | Supplement | Patent on strain level; patent on species level covering processing and claimed indications; | (He, Zhao, et Li 2021; Langella et al. 2021; Seo, Shin, et Lee 2020; Medicine 2018) |
| *Faecalibacterium prausnitzii* | NGP, Human feces | Butyrate production; anti-inflammatory metabolite profile | Amelioration of colitis, protection in Crohn’s disease animal models | Early-phase safety study limited efficacy data | Not commercialized | N/A | Patent on strain level; patent on indications | (Cui et al. 2022; C.-H. Lai, Lin, Huang, et al. 2022; H.-C. Lai, Lin, Chen, et al. 2022; Lin et al. 2024; Qin et al. 2025; Wu et al. 2020) |
| *Parabacteroides goldsteinii* | NGP, Healthy human feces | Bile acid metabolism modulation; anti-inflammatory LPS characterization | Amelioration of colitis, attenuation of COPD and hepatic inflammation in mice and piglets | No human trials to date | Not commercialized | N/A | Patent on strain level; patent on indications | (Holleboom, Nieuwdorp, et De Vos 2025; Attaye et al. 2025; Attaye et al. 2024; Gilijamse et al. 2020) |
| *Anaerobutyricum soehngenii* | NGP, Infant feces | Butyrate production; carbohydrate metabolism | Improved insulin sensitivity and blood pressure in metabolic disease models | Pilot human trials: improved glycemic control and metabolic markers | Not commercialized | N/A | Limited strain-level patent; patents on indication, composition, and production method | (Attaye et al., 2025; Attaye et al., 2024; Gilijamse et al., 2020; Holleboom et al., 2025) |

**Supplementary Table S3. Changes in Probiotic Viability with and without Encapsulation**

| Encapsulation Technique | Target Strain | Condition | Without Encapsulation | With Encapsulation | Improvement | References |
| --- | --- | --- | --- | --- | --- | --- |
| Alginate + POPL | *Lcb. rhamnosus* (8 strains) | pH 2, 2h | 10^4^ CFU/mL | 10^5^ CFU/mL | 10-fold | (Ding et Shah 2009) |
| Alginate | *L. bulgaricus* L2 | Freeze-drying | Not reported | 100% | - | (Dianawati, Mishra, et Shah 2016) |
| Trehalose formulation | *Lcb. rhamnosus* GG | Spray drying | 5-log CFU/g loss | 0. 12-0.26 log CFU/g loss | ≥4.7log CFU/g | (Broeckx et al. 2017) |
| WPC + Pullulan + Trehalose | *L.plantarum* | Spray drying | - | 12.22 Log CFU/g | Survival rate85.12% | (Sun et al. 2020) |
| Micellar casein + sucrose | *Lpb. plantarum* CNCM I-4459 | Freeze-drying | 4-log CFU/mL loss | 0. 4 log CFU/mL loss | 3.6log CFU/mL | (Bodzen et al. 2021) |
| 1.5 % kelp nanocellulose | *L. paracasei* | Simulated digestion environment | 3.7-log CFU/g loss | 1.65 log CFU/g loss | 2-fold | (K. Wang et al. 2022) |
| Tuna oil encapsulation | *Lcb. casei* 431 | Spray drying  Freeze-drying | 38%  70% | 55%  80% | 17%  10% | (Eratte et al. 2015) |
| Goat milk matrix | *B.* BB-12 | Storage at 25°C for 120 days | 6~7 -log CFU/g loss | 4-log CFU/g loss | >100-fold | (Verruck et al. 2019) |
| Alginate + chitosan | *Lm. reuteri*  SW-23 | SGF for 120 min SIF for 180 min | 52.7% | 84.3% | 1.6-fold | (Parsana, Yadav, et Kumar 2023) |
| polymeric matrix | *L. helveticus* | Spray drying  pH4.5, 3 | - | 99%  98% | - | (Barro et al. 2024) |

POPL = Poly-L-lysine,

WPC: Whey Protein Concentrate,

SGF: simulated gastric fluid (pH 1.5),

SIF: simulated intestinal fluid (pH 7.4),

polymeric matrix: milk powder / maltodextrin / trehalose / FOS / starch).

**Supplementary Table S4. Comparative Overview of Regulatory Frameworks for Probiotics by Country**

| **Category** | **Korea** | **China** | **USA** | **EU** | **Japan** |
| --- | --- | --- | --- | --- | --- |
| **Common Features** | 1. Approval determined through pre-market review 2. Disease-related claims not permitted 3. Functionality must be supported by in vitro, animal, and human studies 4. Safety assessments recommended: hemolytic activity, intestinal toxin production, presence of toxic/pathogenic genes, history of infection, antibiotic resistance, WGS, colonization and metabolic characteristics | | | | |
| **Regulatory Authority** | MFDS | NMPA | FDA | EFSA | CAA |
| **Regulatory System** | NI / IAI | Registration / Notification -based System | GRAS / NDI | QPS / NHCR | FOSHU / FFC |
| **Regulatory Scope** | NI: review based on established standards; IAI: functionality and safety assessment | Registration: evaluation of functionality and safety; Filing: simplified notification | Generally regulated as conventional food or dietary supplement | QPS: safety review; NHCR: evaluation of function claims | FOSHU: official review of functionality and safety; FFC: self-substantiated, literature-based functional claim |
| **Product Category** | Healthy functional food | Healthy food | Dietary supplement / food | Conventional food / functional food | FOSHU / FFC |
| **Approval Unit** | NI: species level,  IAI: strain level | Strain level | Strain level | Strain level | △ Primarily product-based |
| **Restrictions on Claims** | Only standardized phrases allowed | Only approved claims may be displayed | Only structure/function claims permitted, expressed as indirect phrases such as “supports” or “helps maintain”; no disease claims allowed | Only standardized phrases permitted | Claims like “helps maintain/improve ○○” allowed |
| **Evaluation of Transferable Resistance Genes** | △ If required | △ If required | △ If required | ○ Mandatory | △ If required |
| **Metabolic Assessment (D-lactate, BSH)** | ○ Conducted | △ Functionality-focused | △ Functionality-focused | △ Functionality-focused | △ Functionality-focused |
| **Acute Toxicity Test (Single Dose)** | X Generally exempt | ○ Conditionally required | X Generally exempt | X Generally exempt | ○  Conditionally required |
| **Repeated-Dose Toxicity Test (e.g., 28-day)** | X Generally exempt | ○ Conditionally required | X Generally exempt | X Generally exempt | ○  Conditionally required |
